# Baboon Envelope Pseudotyped “Nanoblades” Carrying Cas9/gRNA Complexes Allow Efficient Genome Editing in Human T, B, and CD34^+^ Cells and Knock-in of AAV6-Encoded Donor DNA in CD34^+^ Cells

**DOI:** 10.3389/fgeed.2021.604371

**Published:** 2021-02-09

**Authors:** Alejandra Gutierrez-Guerrero, Maria Jimena Abrey Recalde, Philippe E. Mangeot, Caroline Costa, Ornellie Bernadin, Séverine Périan, Floriane Fusil, Gisèle Froment, Adriana Martinez-Turtos, Adrien Krug, Francisco Martin, Karim Benabdellah, Emiliano P. Ricci, Simone Giovannozzi, Rik Gijsbers, Eduard Ayuso, François-Loïc Cosset, Els Verhoeyen

**Affiliations:** ^1^CIRI–International Center for Infectiology Research, Inserm, U1111, Université Claude Bernard Lyon 1, CNRS, UMR5308, Ecole Normale Supérieure de Lyon, Université Lyon, Lyon, France; ^2^Laboratory of Lentiviral Vectors and Gene Therapy, University Institute of Italian Hospital, National Scientific and Technical Research Council (CONICET), Buenos Aires, Argentina; ^3^Université Côte d'Azur, INSERM, Nice, France; ^4^Centre for Genomics and Oncological Research (GENYO), Genomic Medicine Department, Pfizer/University of Granada/Andalusian Regional Government, Granada, Spain; ^5^Laboratory of Biology and Modeling of the Cell (LBMC), Université de Lyon, Ecole Normale Supérieure de Lyon (ENS de Lyon), Université Claude Bernard, Inserm, U1210, CNRS, UMR5239, Lyon, France; ^6^Laboratory for Viral Vector Technology & Gene Therapy, Department of Pharmaceutical and Pharmacological Sciences, Faculty of Medicine, Katholieke Universiteit Leuven, Leuven, Belgium; ^7^KU Leuven, Department of Microbiology, Immunology and Transplantation, Allergy and Clinical Immunology Research Group, Leuven, Belgium; ^8^INSERM UMR1089, University of Nantes, Centre Hospitalier Universitaire, Nantes, France

**Keywords:** hematopoietic stem cells, T cell, B cell, gene editing, CRISPR/Cas9, nanoblade, immunotherapy, gene therapy

## Abstract

Programmable nucleases have enabled rapid and accessible genome engineering in eukaryotic cells and living organisms. However, their delivery into human blood cells can be challenging. Here, we have utilized “nanoblades,” a new technology that delivers a genomic cleaving agent into cells. These are modified murine leukemia virus (MLV) or HIV-derived virus-like particle (VLP), in which the viral structural protein Gag has been fused to Cas9. These VLPs are thus loaded with Cas9 protein complexed with the guide RNAs. Highly efficient gene editing was obtained in cell lines, IPS and primary mouse and human cells. Here, we showed that nanoblades were remarkably efficient for entry into human T, B, and hematopoietic stem and progenitor cells (HSPCs) thanks to their surface co-pseudotyping with baboon retroviral and VSV-G envelope glycoproteins. A brief incubation of human T and B cells with nanoblades incorporating two gRNAs resulted in 40 and 15% edited deletion in the Wiskott-Aldrich syndrome (WAS) gene locus, respectively. CD34^+^ cells (HSPCs) treated with the same nanoblades allowed 30–40% exon 1 drop-out in the WAS gene locus. Importantly, no toxicity was detected upon nanoblade-mediated gene editing of these blood cells. Finally, we also treated HSPCs with nanoblades in combination with a donor-encoding rAAV6 vector resulting in up to 40% of stable expression cassette knock-in into the WAS gene locus. Summarizing, this new technology is simple to implement, shows high flexibility for different targets including primary immune cells of human and murine origin, is relatively inexpensive and therefore gives important prospects for basic and clinical translation in the area of gene therapy.

## Introduction

Gene-editing approaches aim at directly manipulating the genome allowing gene disruption, gene correction, or transgene integration at a precise endogenous genomic locus. In contrast to ectopic gene expression, gene editing has the advantage of allowing a spacio-temporal and thus physiological regulation of transgene expression (Gilbert et al., 2014; Antony et al., 2018; Kuo et al., 2018). An additional advantage over gene addition using integrating viral vectors is that gene editing avoids insertional mutagenesis and gene silencing. Thus, precise genetic manipulation of cells provides unpreceded opportunities for research (Tothova et al., 2017; Chen et al., 2018; Ting et al., 2018) and therapeutic applications (Lombardo and Naldini, 2014; De Ravin et al., 2017; Diez et al., 2017; Kuo et al., 2018; Gentner and Naldini, 2019). Gene editing is based on the induction of DNA double-strand breaks (DSBs) at a specific site in the genome by endonucleases. There are various specific engineered nucleases used as gene editing tools such as zinc finger nucleases (ZFN), transcription activator-like effector nucleases (TALENs), and more recently clustered regularly interspaced short palindromic repeats (CRISPR)/associated protein 9 (Cas9) (Jinek et al., 2012; Gaj et al., 2013; Osborn et al., 2020). The most frequent DNA repair pathway that takes place after DSB is non-homologous end-joining (NHEJ). In this case DNA ends are fused without a repair template and this leads to insertion or deletion of nucleotides, often introducing frame shift mutations, totally or partially blocking gene transcription and translation (Doudna and Charpentier, 2014; Sander and Joung, 2014; Shalem et al., 2014). In contrast, homology-directed repair (HDR) results in complete gene correction by homologous recombination with the sister chromatid or delivery of a donor DNA repair template. The DSB induced by endonucleases at a specific locus can be sealed by HDR when an exogenous DNA template is provided carrying homology arms to the site of the DSB. This template is provided either by integration-deficient lentiviral vectors (IDLVs), recombinant adeno-associated viruses serotype 6 (rAAV6) or by electroporation of single-stranded DNA, or oligonucleotides (ODN) (Hendel et al., 2015a; Wang et al., 2015; Antony et al., 2018). However, since HDR is restricted to the S/G2 phase of the cell cycle, gene modification in primary cells remains a challenge for the scientific community.

In particular, the bacteria-originated CRISPR/Cas9 system has revolutionized the methodology to produce knock-out and knock-in genome editing due to its high specificity, activity and easy design to perform efficient gene editing in cell lines but also in primary cells (Chen et al., 2018; Daniel-Moreno et al., 2019; Hartweger et al., 2019; Moffett et al., 2019). The CRISPR/Cas9 component can be introduced in the cell of interest using different methods, e.g., by using CRISPR/Cas9 encoding retroviral vectors (Heckl et al., 2014) or plasmids (Mandal et al., 2014) and RNAs (Hendel et al., 2015a) encoding for these components introduced by electroporation. Currently though, the method of choice is electroporation of ribonucleoproteins (RNPs), incorporating guide RNA(s) (gRNA), and Cas9 proteins to obtain efficient gene editing in primary human T and B cells and HSPCs (Bak et al., 2018; Wu et al., 2018; Hultquist et al., 2019). This offers a major advantage since the Cas9/gRNAs are only transiently present in the cell, thereby avoiding insertional mutagenesis as reported for integrative vectors (Howe et al., 2008; Patiroglu et al., 2016), implying a safety benefit essential for clinical applications. CRISPR/Cas9 applications cover various fields in biotechnology, biological investigation and human medicine (Gaj et al., 2013; Gupta and Musunuru, 2014). Here we focus on the value of this tool for genome editing in primary gene therapy targets such as T, B and hematopoietic stem and progenitor cells (HSPCs).

Anti-cancer strategies have been revolutionized since the invention of TCR engineered T-cell and chimeric antigen receptor T-cell (CAR-T) therapy. CAR-T cell therapy involves changing a patient's own immune cells to augment the immune reponse to cancer cells (June et al., 2015). Along with CD19 CAR-T cells for B cell malignancies (Porter et al., 2011; Kochenderfer et al., 2012), other CAR-T cells are under evaluation for hematological malignancies (HM) directed against CD5, CD33, CD70, CD123, CD38, and B cell maturation antigen (BCMA) (Townsend et al., 2018). However, CAR T cell immunotherapy is associated with toxicities, exhaustion, immune suppression, lack of long-term persistence, and low CAR T-cell tumor infiltration. Major efforts to overcome these hurdles are currently on the way (Mhaidly and Verhoeyen, 2020). This involves gene editing-mediated knockouts of immune checkpoint regulators such as PD-1, the endogenous TCR and histocompatibility leukocyte antigen (HLA) complex to avoid the graft-versus-host-disease (GvHD) and to generate universal allogeneic CAR-T cells (Ren et al., 2017). Thus, gene editing to generate therapeutic T cells permits the immunotherapy field to move forward quickly.

B cells are also interesting targets for gene editing given their involvement in B-cell dysfunctions, autoimmune diseases and infectious diseases. Indeed, B cells have the potential to induce specific immune activation. Their downstream effectors plasmablasts and plasma cells are specialized antibody-secreting cells and central to humoral immune response (Radbruch et al., 2006; Forthal, 2014). Long-lived plasma cells (LLPCs) can persist a lifetime and assure a continuous supply of serum antibodies (Amanna and Slifka, 2010). Primary B cells and plasma cells were engineered to produce therapeutic antibodies and proteins, such as antibodies against hepatitis C, anti-HIV broadly neutralizing antibodies, and the human clotting factor IX (FIX) (Luo et al., 2009; Fusil et al., 2015; Vasileva et al., 2015; Levy et al., 2016; Hung et al., 2018; Voss et al., 2019). To reprogram B cells for ectopic antibody expression, it would be advantageous to include the transition from the B-cell receptor (BCR) form to secreted immunoglobulins (Ig). To achieve this goal, genome editing in B cells can place the ectopic anti-body expression under the control of endogenous regulatory elements. More recently, CRISPR/Cas9 was used for precision editing in primary human B cells by using Cas9 mRNA or protein combined with chemically modified gRNAs (Hendel et al., 2015a; Johnson et al., 2018). This method was combined with an rAAV6 vector providing the HDR donor template to obtain efficient knock-in in primary B cells (Hung et al., 2018; Johnson et al., 2018; Feng et al., 2019). In contrast to T cells, B cells have received little attention for therapeutic gene editing purposes. Recently though, reprogramming of B cell antigen specificity to specific pathogens has been successful to protect against infections or to secrete anti-PD1 for immune check point inhibition using CRISPR/Cas9 technology (Moffett et al., 2019; Voss et al., 2019; Luo et al., 2020).

Hematopoietic stem cells are “the gene therapy target cells of choice” for cure of many genetic diseases since they will pass on the gene correction in the stem cell to all derived blood lineages. Using lentiviral gene transfer in HSCs, X-linked Severe Combined Immunodeficiency (SCID-X1) gene therapy led to 100% survival rates and over 80% efficiency (Gaspar et al., 2011). However, gene therapy using integrative vectors in HSCs is associated with safety concerns since the integration profiles of these vectors can give rise to genotoxicity or dysregulated transgene expression as detected in the SCID-X1 trials (Demeulemeester et al., 2015), Wiskott-Aldrich syndrome (WAS; Boztug et al., 2006) and X-linked chronic granulomatous disease (X-CGD, (Grez et al., 2011). For this reason, the emerging gene therapy approach of choice for HSCs is gene editing. The correction of genes at their endogenous locus in HSCs can potentially define a safer curative strategy for hematological diseases without the risk of insertional mutagenesis and assure tightly regulated expression of the transgenes by endogenous regulatory elements in all the hematopoietic cell lineages. Schiroli *et al*. achieved functional gene correction for the interleukin-2 receptor gamma (IL2RG) in HSCs from SCID-X1 using ZFN nucleases and an AAV6 vector encoding for a donor DNA repair template (Schiroli et al., 2017). Pavel-Dinu et al. used a CRISPR/Cas9/AAV6-based strategy to introduce the correcting cDNA into the genome under the control of the IL2RG promoter in HSCs (Pavel-Dinu et al., 2019). This approach has the benefit of maintaining the cell intrinsic expression pattern, thereby reducing the likelihood of side effects and may represent therefore a new therapeutic opportunity for SCID-X1 patients. Kuo et al. also used the CRISPR/Cas9 platform to correct another primary immunodeficiency, the X-linked hyper IgM syndrome, by introducing a normal copy of the CD40L cDNA downstream of the endogenous promoter (Kuo et al., 2018). Further, De Ravin et al. used CRISPR/Cas9-based gene editing to obtain successful gene repair of HSCs from GCD patients, by confining transgene expression to the myeloid lineage (De Ravin et al., 2017). Gene-editing strategies for β-hemoglobinopathies (β-thalassemia and sickle cell disease) have rather focused on disruption of silencing factors and regulators such as BCL11A to induce *de novo* expression of fetal hemoglobin (Canver et al., 2015; Antoniani et al., 2018; Martyn et al., 2018). Additionally, HIV infection is one of the most studied diseases using gene editing therapy approaches (Mandal et al., 2014). Strategies based on NHEJ as evoked here for β-hemoglobinopathies and HIV are attractive since NHEJ events occur more frequently in HSCs than HDR, which requires HSCs to cycle (Antony et al., 2018).

For the previously mentioned studies, different methods to deliver the gene editing tools such as electroporation, adenoviruses, AAVs, and lentiviral vectors (LVs) have been used, conferring different degrees of efficiency, toxicity, and off-target effects. Ideally, perfect gene editing tool delivery be fast, precise, non-toxic, and associated with low off-target effects. Recently, we described a vehicle for Cas9/gRNA by which the ribonucleoprotein (RNPs) are packed into a virus-like particle (VLP) from a murine leukemia virus (MLV), called “nanoblade” (Mangeot, 2019; Mangeot et al., 2019). These nanoblades contain Cas9 protein associated with gRNAs and are devoid of a viral genome, which allows thus a transient and rapid RNP delivery into the target cells. We previously have shown that these nanoblades were able to induce DSB more rapidly and efficiently than other delivery methods and they were able to deliver their cargo not only to immortalized cells but also to primary fibroblast and induced pluripotent stem cells (Mangeot et al., 2019). More interestingly, since these are viral-vector-derived particles, they carry an enveloped vector capsid and can be pseudotyped as their counterpart viral vectors with different envelope glycoproteins (gps). We have previously shown that the baboon endogenous virus (BaEV) envelope gp incorporated into a LV, allowed efficient cell entry into human T, B and HSPCs (Girard-Gagnepain et al., 2014; Levy et al., 2016; Bernadin et al., 2019). Here we evaluated if the BaEV envelope gps was as able to confer efficient nanoblade attachment to and fusion with the target cell to release the Cas9-sgRNA complexes they incorporated into relevant human T, B cells and HSPCs and permit efficient gene editing in these primary target cells.

## Materials and Methods

### Plasmids

To construct the GagMLV-CAS9 fusion, sequential insertions of PCR-amplified fragments in an expression plasmid harboring the human cytomegalovirus early promoter (CMV), the rabbit beta-globin intron and polyadenylation signals were performed. For the construction of the MA-CA-NC sequence from Friend Murine Leukemia virus (Accession Number: M93134) the MA/p12 protease-cleavage site (9 aa) and the Flag-nls-spCas9 (*Streptococcus pyogenes* Cas9) amplified from pLenti CRISPR were fused (Mangeot et al., 2019). HIV-CAG-CAS9 (KLAP 229) was constructed by replacing the MA/CA/NC sequence from MLV in BIC-GAG-CAS9 (Addgene #119942) by MA/CA/NC PCR amplified from the HIV sequence (NL4-3) using XhoI and AgeI sites. A protease cleavage site (KARVLAEAMS corresponding to MA/CA HIV-protease site) was inserted upstream the flag-CAS9sp sequence. BaEVRless envelope glycoproteins were previously described (Girard-Gagnepain et al., 2014). All envelope glycoproteins were expressed in the phCMV-G expression plasmid (Maurice et al., 2002).

The cassette containing SFFV-GFP DNA flanked by the WASP gene 3′ and 5′ homologous arms was excised from pDonor-SFFV-GFP plasmid by Sbf1/Pac1 digestion, blunted and cloned into plasmid vector pAAV-MCS-spA (Stratagene), that was previously digested by Pst1/Mfe1 and restriction sites were blunted. The resulting plasmid pAAV-SFFV-GFP contained the ITR2 sequences from AAV serotype 6 flanking the donor DNA cassette.

### Cell Lines

The HEK293T cells (CRL11268; American Type Culture Collection; Rockville, MD) were maintained in Dulbecco's Modified Eagle's Medium (DMEM, Invitrogen, Edinburgh, Scotland) supplemented with 10 % Fetal Bovine Serum (FBS) (PAA Laboratories GmbH, Austria) and penicillin/streptomycin (Gibco, Invitrogen, Auckland, New Zeeland). The human erythroleukaemic cell line K562 (ATCC, Manassas, VA; CCL-243) and the Raji cell line (ATCC; Manassas, VA; CCL-86) were maintained in RPMI 1640 media (Gibco-BRL, Middlesex, UK), supplemented with 10% FBS and penicillin/streptomycin.

### Nanoblade Production

Nanoblades particles were generated by transient transfection of HEK293T cells using CaPO_4_ method. For MLV-derived nanoblade production, 3 μg of Cas9-MLV-gag encoding plasmid (BicCas9) was added. For HIV-derived nanoblades, 3 μg of Cas9-HIV-gag encoding plasmid (KLAP229) was added. Five micro gram of either BaEVRLess (BRL) or VSVG envelope gp-encoding plasmids were transfected for BRL or VSVG gp pseudotyping of nanoblades. For co-pseudotyping of nanoblades with BRL and VSVG, 2 μg of each envelope encoding plasmid was used. Three micro gram of each plasmid coding for a gRNA (301-agcctcgccagagaagacaa and 305-gatgcttggacgaaaatgct) was added as well as 3 μg of the HIV or MLV gag-pol encoding plasmid either for HIV- or MLV-based nanoblade production, respectively. Medium was replaced by Optimen supplemented with Hepes (Gibco, Invitrogen, Auckland, New Zealand) and penicillin/streptomycin 18 h post-transfection. Nanoblades were harvested 48 h post-transfection, centrifuged and filtered through 0.45 μm. Low speed concentration of the nanoblades was performed overnight at 3,000 *g* and 4°C. The concentrated nanoblades were collected the following morning and stored at 4°C.

### Cas9 Quantification in the Nanoblades by ELISA

Recombinant Cas9 (New England Biolabs, USA) was used to generate a standard curve (20μM, 6 serial dilutions of 1/2), while the nanoblade supernatants were diluted 1/200 and 1/400. The dilutions were performed in coating buffer (1% Triton) and were then coated onto 96-well-plates by incubation overnight at 4°C. The following day, the wells were incubated with washing buffer (PBS/0.05% Tween) and blocked with PBS/0.05%Tween/3%BSA (Sigma). Subsequently the wells were washed and the primary anti-Cas9 antibody (Cas9-7A9-3A3, 14697P; Cell signaling Technology, Inc., USA) was added at 1/1,000 dilution in PBS/3% BSA, and incubated at RT for 1 h, while shaking. Before and after 1 h incubation with a secondary anti-mouse HRP (F6009-X639F South biotech, USA) diluted 1/10,000 in PBS/3%BSA, a wash-step was performed. Finally, the mixed TMB substrate solution, containing HRP substrate was added for 20 min (Bethyl, Inc., Texas, USA). Stop reaction was added in each well and protein was measured at 450 nm in a Multiskan FC (Thermo Scientific).

### Production of AAV6 Vectors

The pAAV-SFFV-GFP contained the ITR2 sequences from AAV serotype 6 flanking the donor DNA cassette and was used to produce recombinant AAV2/6 vectors as described previously (Ayuso et al., 2010). Briefly, HEK293 cells in CellStack-5 chambers (CS5) were transfected with two plasmids (pDG6 containing rep2cap6 sequences and adenovirus genes, and the vector plasmid pAAV-SFFV-GFP) by the calcium phosphate precipitation method, cells were harvested 72 h post-transfection by centrifugation. The cell pellet was resuspended in TBS buffer and AAV particles were extracted by freeze-thaw cycles. Upon centrifugation, the supernatant was PEG-precipitated, then purified by double CsCl density gradient ultracentrifugation, and finally formulated in 1 × DPBS containing Ca^2+^ and Mg^2+^ through dialysis in Slide-A-Lyzer 10 K cassettes (Thermo Scientific, Illkirch, France). Vector genomes were titrated by quantitative PCR as followed: 3 μl of purified AAV vectors were treated with 4U of DNase I (Sigma-Aldrich) in DNase buffer (13 mM Tris pH7.5, 0.12 mM CaCl_2_, and 5 mM MgCl_2_) for 45 min at 37°C. Then, DNase I-resistant nucleic acids were purified by the NucleoSpin RNA Virus kit (Macherey-Nagel, Hoerdt, France), and vector genomes were quantified by TaqMan qPCR in Premix Ex Taq probe qPCR master mix (TaKaRa Bio, Saint-Germain-en-Laye, France). Primers were targeted to ITR2 sequence and the standard curve was prepared as described previously (D'Costa et al., 2016).

### Primary Lymphocyte Isolation

Peripheral blood samples were obtained from healthy donors after informed consent and approval was obtained by the ethical committee of the hospital according to the Helsinki declaration. PBMCs were isolated using Ficoll gradient (Sigma-Aldrich, St Louis, MO). CD19^+^ B cells and CD3^+^ T cells were purified by negative selection using the B cell isolation Kit II (Miltenyi Biotec) for CD19^+^ B cells and the human Pan T cells isolation kit (Miltenyi Biotec) for the CD3^+^ T cells following manufacturer's instructions followed by separation through the Automacs pro-separator (Miltenyi Biotec). Purity of isolated B and T cells was monitored using anti-hCD19APC and anti-hCD3PE antibodies (Miltenyi Biotec), respectively, and was analyzed by flow cytometry (MACSQuant VYB, Milteny Biotech).

### CD34^+^ Cells Isolation

Umbilical cord blood (CB) samples from full-term pregnancies (provided by Lyon Sud Hospital, Lyon) were collected in bags containing anti-coagulant after informed consent of donors and approval was obtained by the ethics committees of the hospitals according to the Helsinki Declaration. Low-density cells were separated by Ficoll gradient (Sigma-Aldrich, St Louis, MO). CD34^+^ purification was performed by positive magnetic cell separation using the Automacs pro-separator (Miltenyi Biotech) after staining of the cells with the human CD34^+^ MicroBead Kit (Miltenyi Biotec). Purity of the selected CD34^+^ cell fraction was evaluated by FACS analysis (FACSCanto, BD) with APC-conjugated anti-CD34 antibody (Miltenyi Biotech). Cells were frozen in FCS 10% DMSO for later use.

### Transduction of Cells With Nanoblades

For nanoblade transduction into cell lines: 2E5 293T, K562, or Raji cells were plated in 6- (293T) or 24-well plates (K562, Raji) and nanoblades, equivalent to 4 μm of Cas9 protein, were added. Cells were pelleted 48 h post-transduction for subsequent DNA extraction and PCR. For nanoblade transduction of lymphocytes: freshly isolated unstimulated lymphocytes were seeded in RPMI 1640 medium (Gibco Invitrogen, Auckland, New Zeland) supplemented with 10% FSC (Lonza, Verviers, Belgium) and penicillin/streptomycin (Gibco, Invitrogen, Auckland, New Zealand). B cells were stimulated for 24 h with 200 ng/ml Pansorbin A [Staph. Protein A (SpA; Sigma)] and 100 ng/ml IL-2. T cells were activated for 3 days with IL-7 (20 ng/ml; Miltenyi Biotech) or stimulated through the TCR using TransAct CD3/CD28 beads (Miltenyi Biotech) supplemented with IL-2 (100 ng/ml) in RPMI medium as previously described (Bernadin et al., 2019). CD34^+^ cells were thawed and seeded in Cellgro medium (Cell genix, Germany) and stimulated with cytokines (human thrombopoietin (hTPO), 20 ng/ml; human stem cell factor (hSCF), 100 ng/ml; human FMS-like tyrosine kinase 3 ligand (hFlt3-L),100ng/ml) for 24 h before incubation with nanoblades. Viability was determined before and after nanoblade incubation (see below).

For nanoblade transduction of primary cells, 1.5 × 10^5^ CD34^+^, T and B cells were seeded in 48-well-plates coated with RetroNectin® (Clontech/Takara; 12 μg/ml PBS according to manufacturer's recommendations) to which nanoblades (4 μm of Cas9 protein), were added. After 8–16 h of incubation with nanoblades, fresh media was added. 8 h later cells were pelleted for DNA extraction. T cells preactivated for 3 days with IL-7 (20 ng/ml; Miltenyi Biotech) and incubated with nanoblades or not as described above, were continued in culture supplemented with rIL-7 or in the presence of TransAct CD3/CD28 beads (Miltenyi Biotech) supplemented with IL-2 (100 ng/ml) in RPMI. Cells were replenished with IL-7 or IL-2, respectively every 3 days. Cell viability (DAPI staining), proliferation rates, gene editing, and phenotyping was performed by FACS for detecting surface expression of CD4, CD8, CD45RA, CD45RO day 3, day 6, and day 10 of culture.

For pro T cell differentiation, CB CD34^+^ cells were cultured in 48-well plates coated with a Dll4 Fc fusion protein (Dll4-Fc, 5 mg/mL; PX9Therapeutics, Grenoble, France). Cultures were initiated at 3 × 10^4^ CD34^+^ cells per well in X-VIVO-20 medium (Lonza, Basel, Switzerland) and supplemented with 20% defined fetal calf serum (Hyclone; Thermo Fisher Scientific, Illkirch, France) and cytokines: hIL-7, hFlt3-L, hSCF, and hTPO (each at 100 ng/mL; Miltenyi Biotech). Nanoblades preincubated with vectofusin (12 μg/mL; Miltenyi Biotech) were added to the pro T cell cultures at day 5 of differentiation for 24 h, then pelleted for DNA extraction and PCR. At day 3, 7, and 14 of culture, the cells were analyzed by flow cytometry for surface expression of CD34, CD7, and CD5 to distinguish the T cell subpopulations.

### Viability

Viability of T, B, and CD34^+^ cells upon nanoblade incubation was determined using Annexin V/ propidium iodide staining and was then analyzed by flow cytometry.

### Quantification of DNA Editing Efficacy

Genomic DNA extraction for cell lines and primary cells was performed using the NucleoSpin tissue kit (Macherey-Nagel, GmBH & Co.) The genomic region flanking the cleavage site targeted by the nanoblades with two gRNAs (301 and 305) was amplified by PCR with the WASFw/WASRv primers (AGGGTTCCAATCTGATGGCG/TTGAGAACTGGCTTGCAAGTCC) or the WAS2Fw/WAS2Rv(ATTGCGGAAGTTCCTCTTCTTACCCTG/TTCCTGGGAAGGGTGGATTATGACGGG). The PCR product was run on a 1% agarose gel. With the WASFw/WASRv primer pair one observed one fragment of 811 bp when no cleavage or only 1 DSB occurred and an additional fragment of 647 bp when the two gRNA (301 and 305) target sites were cut simultaneously in the WAS gene. With the WAS2Fw/WAS2Rv primer pair we observed a 351 bp band when no cleavage or only 1 DSB occurred and an additional fragment of 227 bp was observed when the two gRNA (301 and 305) target sites were cut simultaneously in the WAS gene. The percentage of cleavage was determined by densitometry with FluorChem Sp (Alfpha Inmotech) of the two bands. Note that the gel blots for the PCR products shown in the figures were in some cases overexposed and contrast was increased to better see the two PCR bands but the original unsaturated images were used for quantification.

### Detection of Single gRNA On-target Efficiency

In addition, upon nanoblade incubation, genomic DNA of the cells was isolated and the genomic region flanking the cleavage site targeted by the nanoblades with two gRNAs (301 and 305) was amplified by PCR with the hWASFw/hWASRv primers. The fragment of 811 bp generated using the hWASFw/hWASRv primer pair englobing the two gRNA target sites was separated from the 647 bp band present when both gRNA target sites were cut (Figure 6). The residual single cuts at target sequences for gRNA301 or gRNA305 were evaluated in the 811 pb PCR band by Sanger sequencing using following primers: Fw-target 1 (GCCCAAGCTCAGCCTAACG) for gRNA301 target site and RV-Target 2 (GAAATGCCGGAAGTCCACTGG) for gRNA305 target site. The chromatograms were analyzed by the online tool ICE (https://ice.synthego.com) and TIDE (https://tides.deskgen.com) (Brinkman et al., 2014). ICE analysis allows for +40bp/-40pb INDEL detections while by default the TIDE algorithm allows +10 bp/−10 bp INDEL detection.

### Off-target Detection

Genomic loci that were similar to the gRNA 301 and gRNA 305 target sequence were identified through CRISPR Seek (http://www.bioconductor.org). We selected the two most probable off-target genomic sites for gRNA301 target sequence and the two most probable off target for gRNA 305 target sequence. PCR primers were designed to amplify a 400 bp fragment around the genomic region of these off-target site (See Supplementary Tables 1, 2). Firstly, genomic DNA was extracted from nanoblade-treated cells using the Nucleospin gDNA extraction kit (Macherey-Nagel). Then, 50 ng of genomic DNA was used for PCR amplification. The PCR products were verified for off-target cuts by performing the surveyor assay: PCR products were diluted by a factor 2 and complemented with Buffer 2 (New England Biolabs) to a final concentration of 1X. Diluted PCR amplicons were then heat denatured at 95°C and cooled down to 20°C with a 0.1°C/second ramp. Heteroduplexes were incubated for 30 min at 37°C in presence of 10 units of T7 Endonuclease I (NEB). Samples were finally run on a 2.5% agarose gel or on a BioAnalyzer chip (Agilent) to assess editing efficiency. In parallel, the obtained PCR-products for off-target sites, were purified for Sanger sequencing using a kit (Nucleospin Gel and PCR Clean up kit, Macherey Nagel, ref 740609). Sanger sequencing used the same primers as for the Surveyor assay (Supplementary Tables 1, 2). The obtained sequences were then analyzed for INDELs at the off-target sites using ICE (https://ice.synthego.com) and TIDE analysis (Brinkman et al., 2014).

### Combined Nanoblades and AAV6 Treatment of K562 Cells and Human CD34^+^ Cells

K562 cells and CD34^+^ cells were treated with nanoblades as described above. Together with the nanoblades, the rAAV6 vectors encoding for the donor cassette were added to the cells at indicated MOIs. CD34^+^ cells were pre-stimulated 72 h in Cellgro medium supplemented with cytokines (hTPO, 20 ng/ml; hSCF, 100 ng/ml; hFlt3-L; 100 ng/ml) and seeded on plates coated with RetroNectin® (Clontech/Takara; 12 μg/ml PBS) according to manufacturer's recommendations, prior to nanoblade (4 μmoles of Cas9 protein) and rAAV6 addition. Eight hours later the medium was changed for Cellgro medium supplemented with cytokines and cultured for 48 h. The cells were washed, counted, and used for flow cytometry analysis, pelleted for genomic DNA isolation to confirm stable integration of the donor cassette or seeded in methyl cellulose medium (STEMCELL Technologies) to perform a colony forming cell (CFC) assay according manufacturer's recommendation (Levy et al., 2017). CFCs were analyzed at day 14 of culture for GFP expression and DNA was isolated from these CFCs to confirm stable integration of the donor cassette by PCR.

### Analysis of Stable Integration of Donor Cassette in K562 and CD34^+^ Cells

Genomic DNA isolated from K562 or CD34^+^ cells or CFCs was subjected to PCR with 1 forward primer situated in the endogenous WAS locus, WAS-FW (AGGGGCTCGCTCTGTAATTA) and a reverse primer in the reporter GFP, REV-GFP (AACTTGTGGCCGTTTACGTC).

### Statistical Analysis

We have applied the unpaired *t*-test to compare two sample groups of the experiment which are performed at least three times using for each experiment a different primary cell donor and a different nanoblade preparation.

## Results

### BaEV and VSV-G gp Co-pseudotyped Nanoblades Confer High Level Gene Editing in Cell Lines

**Figure 1:**
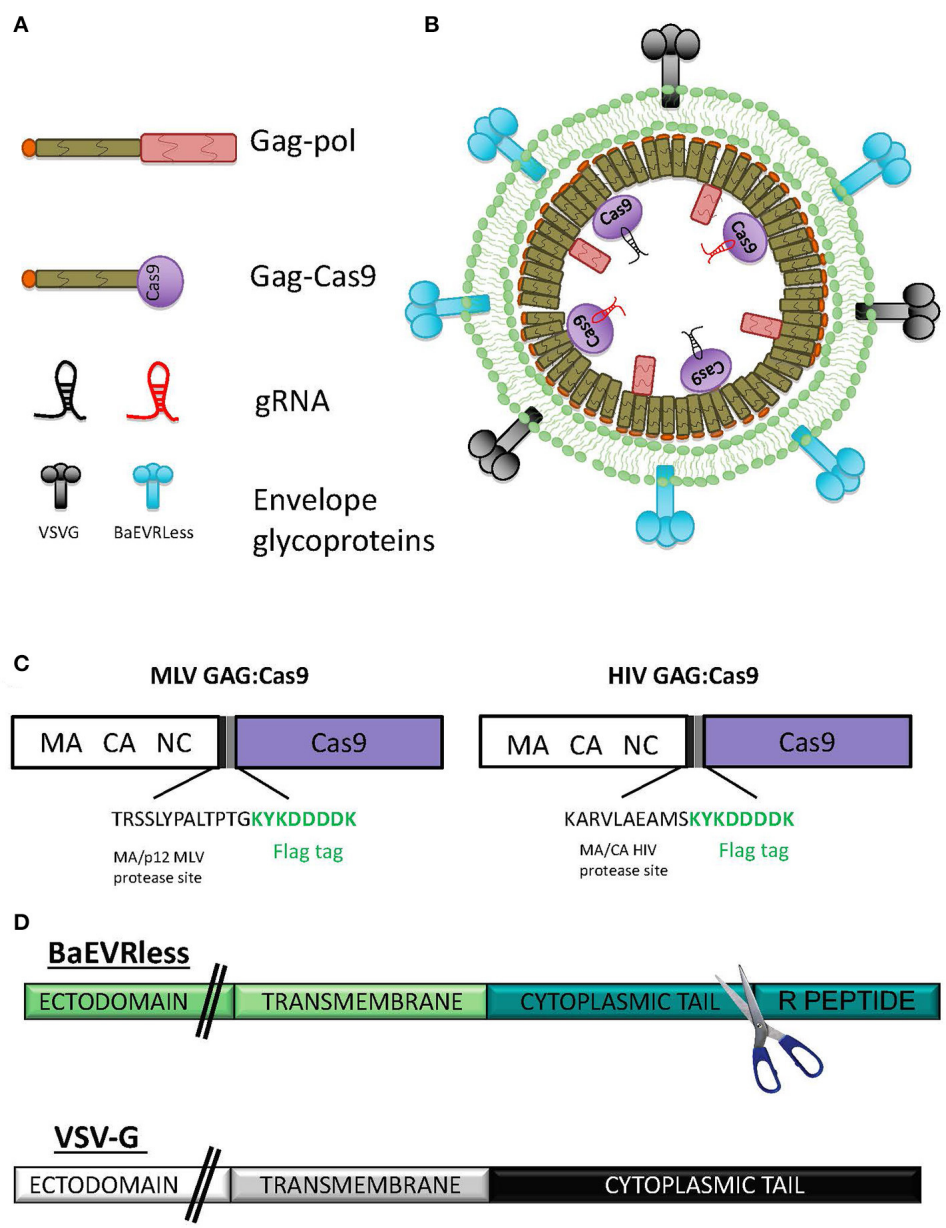
Schematic presentation of nanoblades. **(A)** Representation of the different components of the nanoblades: gag-pol, the gag-Cas9 fusion protein, the different gRNA and the envelope glycoproteins (gps), **(B)** Schematic representation of the assembly of a nanoblade particle, **(C)** Scheme of the MLV GAG:Cas9 fusion (left) and HIV GAG:Cas9 fusion (right) indicating the inserted MLV or HIV protease site followed by a Flag tag. **(D)** Schematic representation of the mutant BaEV envelope gp (BaEVRless) and the VSV-G envelope gp. The R-peptide of the cytoplasmic tail of BaEVwt was deleted resulting in the BaEVRLess mutant gp.

Earlier, we developed the nanotechnology called nanoblades, which are virus-like particles (VLPs) derived from the murine leukemia virus (MLV) (Mangeot et al., 2019). These MLV-based nanoblades are composed of a gag polyprotein fused to a flag-tagged version of *Streptococcus pyogenes Cas9* (Gag-Cas9) and separated by a proteolytic cleavage site borrowed from the MA/p12 MLV junction (Figures 1A–C). These particles can incorporate one or more guide RNAs through association with Cas9. Here, we developed the corresponding HIV-derived nanoblades by fusing Cas9 to the HIV gag protein. To allow release of the Cas9 into the cells, a proteolytic site situated between HIV MA and CA was inserted between Gag and Cas9, which can be cleaved by the HIV protease in the HIV-based nanoblades (Figure 1C). Nanoblade cell entry is conferred by surface display of envelope gps equivalent to pseudotyping of γ-retroviral and LV particles (VSVG, BaEVRless, Figure 1D) (Girard-Gagnepain et al., 2014). To produce the nanoblades, we utilized a protocol similar as used for MLV retroviral or LV production. HEK-293T cells were transfected by the CaPO_4_ method with plasmids coding for MLV or HIV Gag-Cas9, Gag-pol and plasmids coding for one or more single-guide RNA (gRNA) and viral envelope glycoproteins (Figure 1A), which then released the pseudotyped nanoblades in the culture medium (Figure 1B).

**Figure 2:**
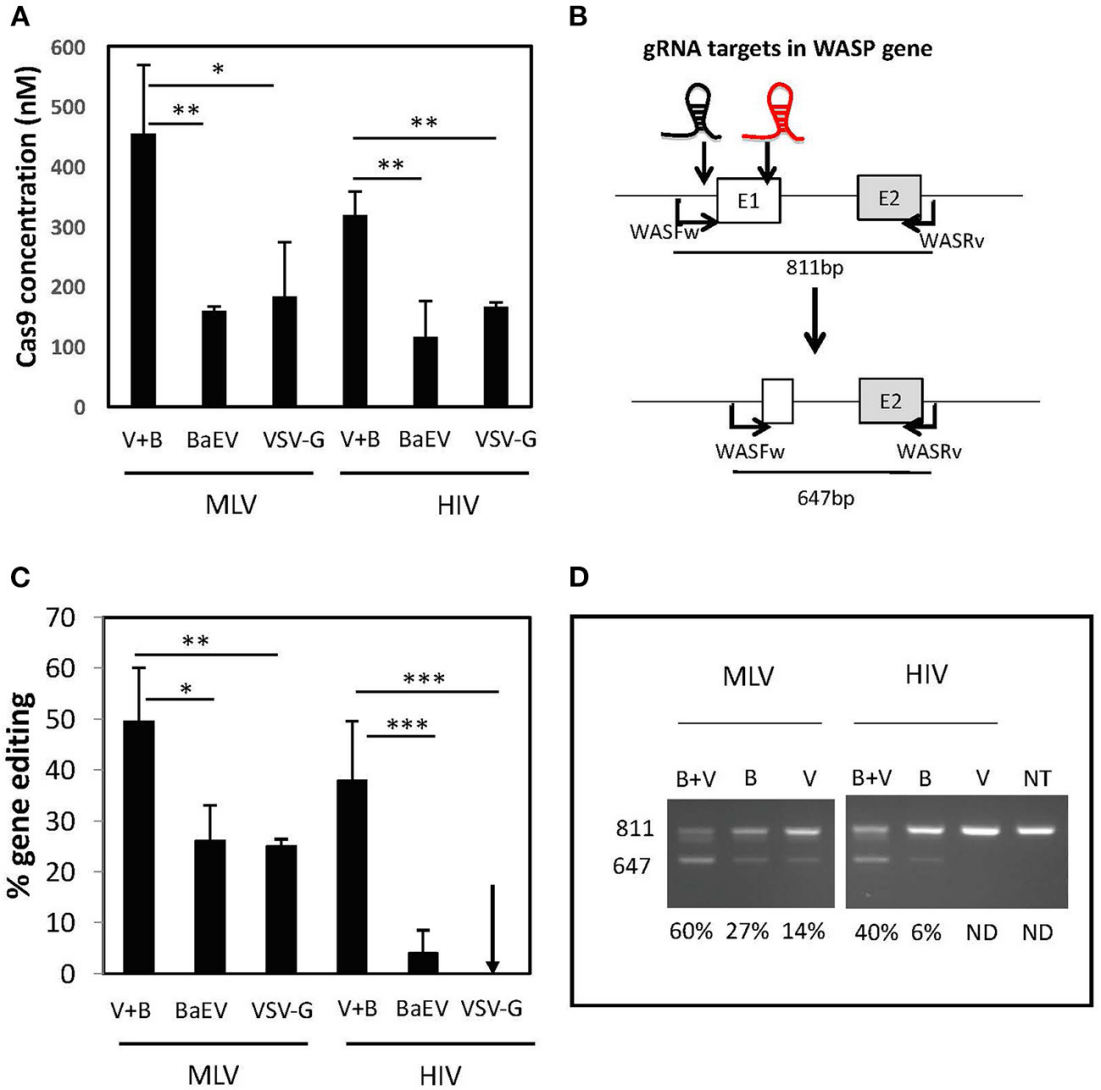
BaEV/VSV-G gp pseudotyped nanoblade-mediated gene editing in cell lines. **(A)** Concentration of Cas9 in nM present in the nanoblades measured by ELISA in MLV and HIV-derived nanoblades co-pseudotyped with BaEV- and VSVG-gps (B + V) or pseudotyped only with BAEV-gp or VSVG-gp (V) (means ± SD, *n* = 3; student *t*-test, *p < 0.05 and **p < 0.01) **(B)** Schematic representation of expected band sizes of WAS PCR product with WASFw/WASRv primers before the cleavage (811 bp), which represent the intact WAS gene and the edited WAS gene after the deletion upon DSB at both gRNA targets (647 bp); the gRNA-301 and−305 target sites are indicated as a black and red gRNA, respectively; **(C)** Graph summarizing percentage of cleavage (right) (means ± SD; MLV B + V *n* = 17, MVL B *n* = 4, MLV V *n* = 5, HIV B + V *n* = 16, HIV B *n* = 7, HIV V *n* = 6; student *t*-test, *p < 0.05, **p < 0.01, and ***p < 0.001). **(D)** Representative electrophoresis gel of the PCR products on genomic DNA from 293T cells transduced with MLV or HIV nanoblades co-pseudotyped with B + V, B, or V gps; quantification of gene editing is indicated for each lane (left).

Previously, it became clear that MLV-based nanoblades co-pseudotyped with VSV-G and BaEV gps were more efficacious for gene editing in cell lines than single gp pseudotyped nanoblades Interestingly, we quantified the amount of Cas9 protein in the nanoblades by ELISA and detected that when both envelopes were present on the MLV-based nanoblades, more than two-fold higher Cas9 protein levels had incorporated than when either of the two envelopes (BaEV or VSVG) was present alone (Figure 2A). Equivalent results were obtained in the context of HIV-based nanoblades (Figure 2A). This observation suggested that the combination of both envelope glycoproteins helped recruiting Cas9 protein and the associated gRNAs into the nanoblades.

To evaluate the efficiency of the MLV- and HIV-based nanoblades, we first targeted the 293T cell line for induction of genomic DSB. We designed two gRNA targeting the exon 1 of the WASP gene, which will result in a 170 bp deletion in the WAS locus. This NHEJ-mediated dropout of exon 1, can readily be detected by PCR using the primer pair, WASFw and WASRv, indicated in Figure 2B. To assess if the higher incorporation of Cas9 also induced more gene editing, we incubated 293T cells with the different MLV- and HIV-based nanoblades displaying either of the two envelope gps (BaEV or VSVG) or each one alone. We isolated genomic DNA and performed a PCR using WASFw and WASRv primers to amplify the WAS gene locus, 48 h post-treatment. Coinciding with higher Cas9 incorporation in MLV nanoblades, we observed that the percentage of gene editing was higher when both envelopes were present (up to a 60% of exon 1 deletion for V+B, Figures 2C,D) as compared to BaEV alone (up to 31% exon1 deletion) and VSV-G alone (up to 25% exon 1 deletion) (Figures 2C,D). HIV-derived nanoblades showed a similar result (Figures 2C,D). Moreover, no toxicity of nanoblades was detected on 293T cells and gene editing was stable (Supplementary Figure 1).

### BaEV and VSV-G gp Co-pseudotyped Nanoblades Permit Efficient Gene Editing in Human T and B Cells

**Figure 3:**
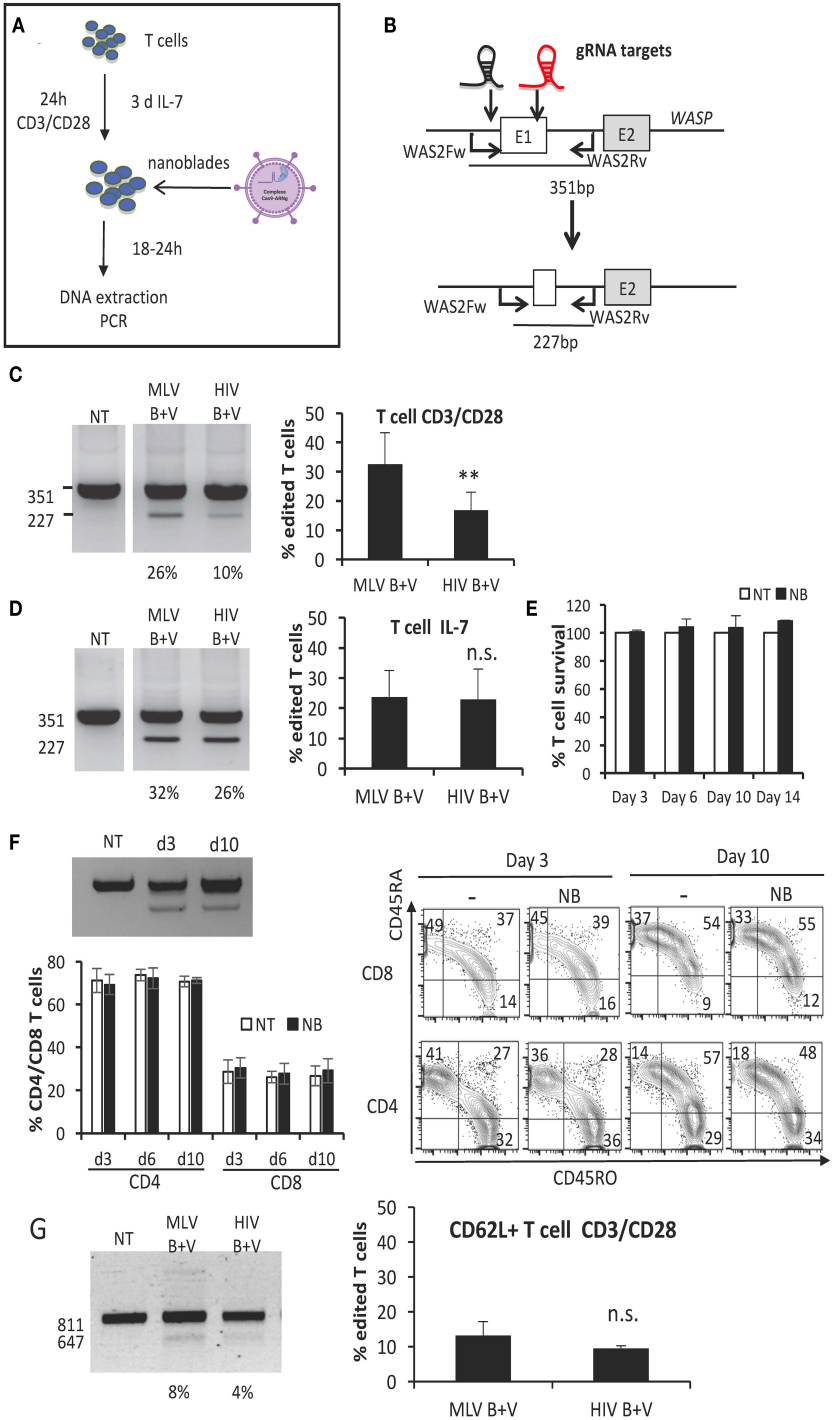
BaEV/VSV-G gp displaying nanoblades allow efficient gene-editing in human T cells. **(A)** Outline of T cell activation and incubation with nanoblades for evaluation of CRISPR/CAS9 gene editing. **(B)** Schematic representation of expected band sizes of the PCR products using the WAS2Fw/WAS2Rv primer pair without cleavage (351 bp) and after gene-edited deletion into the WAS gene (227 bp); gRNA-301 and−305 target sites are indicated as black and red gRNA, respectively. CD3/CD28 activated **(C)** or IL-7 stimulated **(D)** T cells transduced with MLV- or HIV-based nanoblades co-pseudotyped with BAEV- and VSVG-gps (B + V) in the presence of retronectin. **(C,D)** Representative electrophoresis gels of the PCR products using the WAS2Fw/WAS2Rv primer pair; percentage of edited T cells is indicated under each lane (left) and summarizing graph showing percentage of gene editing (right) (means ± SD, MLV B + V *n* = 3, HIV B + V *n* = 3; student *t*-test, ^**^p < 0.01, n.s, not significant). Survival **(E)** and gene editing **(F)** over time are shown for IL-7 stimulated T cells incubated with MLV nanoblades or not (NT) and continued in culture in the presence of IL-7 (means ± SD, NT *n* = 3, NB *n* = 3). Cell survival was determined by DAPI staining and is shown for the nanoblade treated cells (NB) relative to the not treated cells (NT), for which cell survival was set to 100%. **(F)** The percentage of CD4 and CD8 T cells is shown and a representative electrophoresis and flow cytometry plot are shown for CD45RO and CD45RA phenotyping for day 3 and 10 of IL-7 stimulated T cells incubated with MLV nanoblades or not (NT) and continued in culture in the presence of IL-7 (means ± SD, NT *n* = 3, NB *n* = 3; electrophoresis gel and flow cytometry plot: representative of *n* = 3). **(G)** CD3/CD28 activated purified CD62L^+^ T cells transduced with MLV- or HIV-derived nanoblades, co-pseudotyped with BaEV- and VSVG-gps (B + V) in the presence of retronectin. Representative electrophoresis gels of the PCR products using the WASFw/WASRv primer pair (see Figure 2B); the percentage of edited CD62L^+^ T cells is indicated under each lane (left) and graph summarizing percentage of gene editing (right) (means ± SD, MLV B + V *n* = 3, HIV B + V *n* = 3; n.s., not significant).

Since the BaEV and VSV-G gp co-pseudotyped nanoblades, outperformed the single pseudotypes for gene editing in 293T cells, we set out to evaluate them on valuable primary gene therapy targets such as human T and B cells. These cells are not easy to transfect and electroporation of Cas9/gRNA RNPs is toxic to some extent. Importantly, the BaEV envelopes have been shown in the context of lentiviral vectors to allow efficient entry in T cells as well as B cells without affecting their survival (Levy et al., 2016; Bernadin et al., 2019). As depicted in Figure 3A, T cells were pre-stimulated through the T cell receptor (TCR) using anti-CD3/anti-CD28-coated beads or alternatively with the T cell survival cytokine, IL-7, and then transduced with the MLV- and HIV-derived nanoblades loaded with Cas9 associated with the 2 gRNAs directed again exon 1 of WAS (Figure 3B). Following 24 h nanoblade incubation genomic DNA was isolated and gene editing was evaluated by PCR using the WAS2Fw and WAS2Rv primer pair resulting in a 351 bp band when none or only one of the gRNAs target site was cut or a 227 bp band when both target sites were cut simultaneously (Figure 3B). For T cells stimulated through the TCR, we detected up to 40% of genomic deletion with the MLV nanoblades while HIV nanoblades resulted maximum in 25% deletion (Figure 3C). Upon a much milder stimulation with survival cytokine IL7, no difference between the two nanoblade systems (MLV or HIV), was detected resulting in up to 35% of WAS gene deletion (Figure 3D). Of note, we are revealing here by PCR only the cells that were cut at both gRNA target sites simultaneously. We verified stability of gene edting in two different culture conditions for T cells: (1) IL-7 (survival cytokine) culture and (2) CD3/CD28 stimulation inducing proliferation for 10 days. We evaluated for these cultures, gene editing, cell survival and proliferation, as also the % CD4 and % CD8 memory (CD45RO+) and naïve (CD45RA+ cells) over time (Figure 3 and Supplementary Figure 2). We confirmed that there is no short- nor long-term toxic effect of the nanoblades on cell survival, proliferation and that gene editing is stable over time (Figures 3E,F and Supplementary Figure 2). Moreover, no difference in CD4/CD8 T cell ratio and in memory vs. naïve T cell proportions was detected in presence or absence of nanoblades (Figures 3E,F and Supplementary Figure 2).

Another important target cell is the memory T cell, which confers long-term persistence *in vivo* and is characterized by CD62L surface expression. Therefore, we also evaluated gene editing efficiency of nanoblades in sorted CD62L^+^ T cells. Upon incubation with anti-CD3/anti-CD28-coated beads and nanoblade incubation for 24 h CD62L^+^ T cells were collected and gene editing was evaluated by PCR. Again, no significant difference in gene editing (10% on average) between the MLV- and HIV-based nanoblades was detected (Figure 3G).

Other important immature T-cell targets for gene modification are the T-cell progenitors since they may permit long-term T-cell persistence *in vivo* by assuring a continuous output of modified T cells. It was demonstrated that T-cell progenitors can be generated from CD34^+^ hematopoietic stem and progenitor cells (HSPCs) in a feeder-cell-free culture system based on Delta-like Ligand 4 coated on culture plates in the presence of a cytokine cocktail (hSCF, hTPO, and hFlt3-L) mimicking the contact with human thymic tissue (Reimann et al., 2012) (Supplementary Figure 3A). Additionally, we have recently demonstrated that these *in vitro* generated progenitor T cells (pro T cells) are efficiently transduced by BaEV gp pseudotyped LVs (Bernadin et al., 2019). Moreover, these pro T cells were capable of differentiating into mature T cells *in vitro* and accelerating T-cell reconstitution *in vivo* compared with HSPCs (Bernadin et al., 2019). We therefore evaluated the performance of nanoblades in the pro T cells (Supplementary Figure 3A). To distinguish the different T cell populations by flow cytometry, cells were stained with CD7 and CD34 antibodies during differentiation (Supplementary Figure 3B). We distinguished between (1) early lymphoid progenitors (ELP; CD34^+^CD7^−^) and (2) early thymic progenitors (ETP; CD34^+^CD7^+^) and (3) the population of progenitor T cells (Pro T, CD34^+^ CD7^+^). We transduced the cells at day 5 of differentiation and collected the cells 24 h later. WAS gene locus showed the expected deletion (19 and 10%) due to concurrent cutting at both gRNA-target sites when using MLV or HIV nanoblades, respectively (Supplementary Figure 3C).

**Figure 4:**
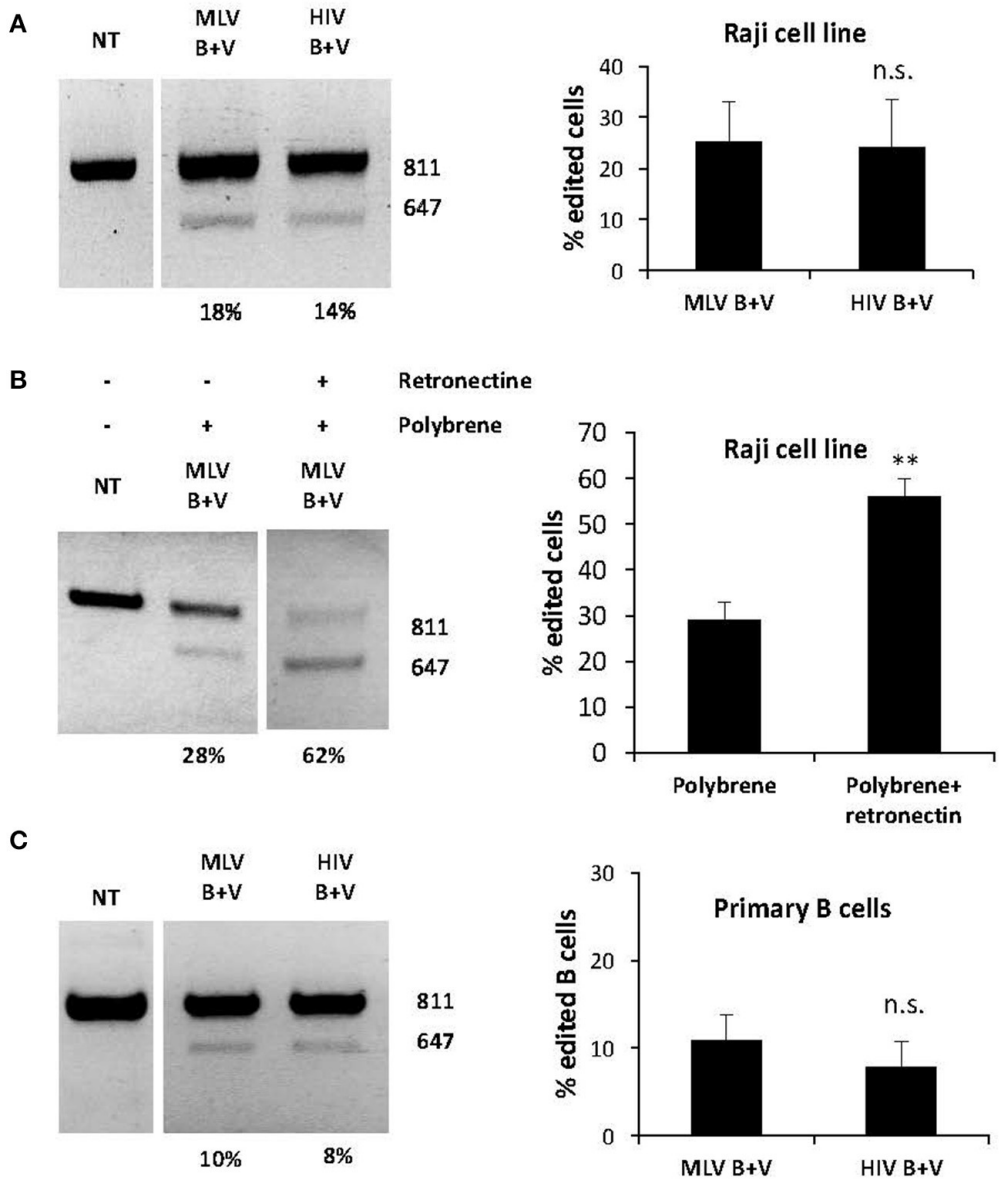
BaEV/VSV-G gp displaying nanoblades allow efficient gene-editing in hB cells. **(A)** Raji cells transduced with MLV- or HIV-based nanoblades co-pseudotyped with BaEV- and VSVG-gps (B + V). Electrophoresis gel of the PCR product using WASFw/WASRv primers with the % gene-editing indicated for each lane (left) and summarizing graph showing percentage of gene editing (right) (means ± SD; MLV B + V *n* = 4, HIV B + V *n* = 5), **(B)** Raji cells were treated with polybrene (PB) or with polybrene and Retronectin (PB+R) and then incubated with MLV nanoblades co-pseudotyped with B + V. Electrophoresis gel of the PCR product using WASFw/WASRv primers with the % gene-editing indicated for each lane (left) Graph showing percentage of gene editing (right) (means ± SD; MLV B + V *n* = 3, HIV B + V *n* = 3; student *t*-test, **p < 0.01, n.s., not significant). **(C)** Primary human B cells preactivated with Pansorbin A and IL-2 were incubated with MLV or HIV nanoblades co-pseudotyped with BaEV- and VSVG-gps (B + V). Representative gel of the PCR product using WASFw/WASRv primers with the percentage of gene-editing indicated for each lane (left) and graph showing percentage of gene editing (right) (means ± SD MLV B + V *n* = 3, HIV B + V *n* = 3; student *t*-test, n.s., not significant).

As mentioned above, B cells are also valuable targets for genetic engineering including gene editing. Before evaluating gene editing using nanoblades in primary human B cells, we treated the Raji B cell line with nanoblades and observed around 25% deletion with both HIV and MLV nanoblades (Figure 4A). Interestingly, when we seeded the Raji cells on Retronectin coated plates and added polybrene a two-fold increase was observed when using both transduction facilitating agents (up to 60% deletion) as compared to using polybrene alone (Figure 4B). Therefore, these agents were applied also during nanoblade incubation of primary B cells. It is generally accepted that human B cells are difficult to transduce with classical VSV-G gp pseudotyped vectors (Amirache et al., 2014). In contrast, LVs pseudotyped with BaEV-LVs easily can reach up to 80% B cell transduction. Therefore, we incubated human B cells pre-stimulated through the BCR with Pansorbin A and IL2, with VSV-G and BaEV gp co-pseudotyped MLV and HIV nanoblades for 24 h. We subsequently confirmed by PCR around 12–15% of DSBs at the two different target sites for the gRNA simultaneously resulting in a deletion of the WAS genomic locus (Figure 4C).

### Nanoblades Allow Efficient Genome Editing in Primary Human HSPCs

Currently, the method of choice to allow efficient gene editing in HSPCs (CD34^+^ cells) relies on CRISPR/Cas9 RNP electroporation. However, this manipulation affects CD34^+^ cell viability. For obvious reasons, this should be avoided since CD34^+^ cells are rare and isolation of sufficient CD34^+^ cells for gene therapy can be a challenging task, especially in the case of BM failures, a family of genetic diseases that affects directly the HSPCs decreasing their numbers with age in the patients (Verhoeyen et al., 2017).

**Figure 5:**
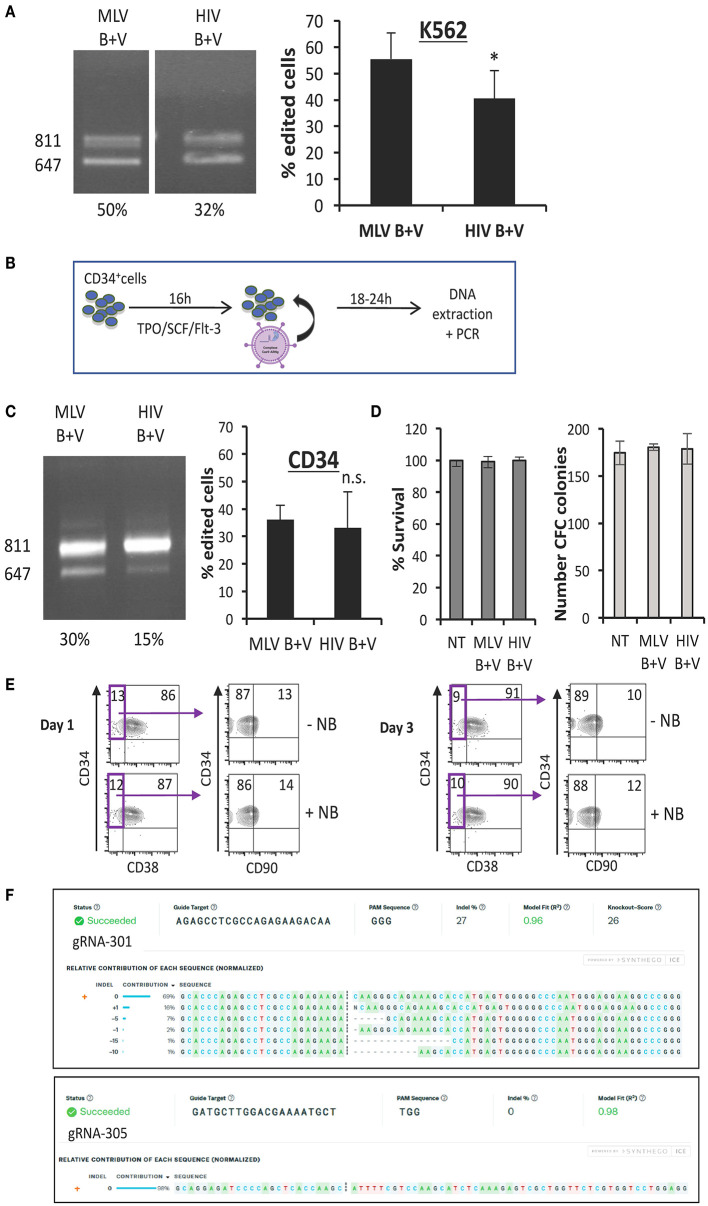
Nanoblades permit efficient gene-editing in human K562 cells and CD34^+^ cells. **(A)** K562 cells transduced with MLV- or HIV-based nanoblades co-pseudotyped with BaEV- and VSVG-gps (B + V). Electrophoresis gel of the PCR product using WASFw/WASRv primers with the % gene-editing indicated for each lane (left) and summarizing graph showing percentage of gene editing (right) (means ± SD; (MLV B + V *n* = 7, HIV B + V *n* = 6; student *t*-test, ^*^p < 0.05). **(B)** Schematic presentation of protocol used for CD34^+^ cells. CD34^+^ cells were activated with cytokines (TPO/SCF/Flt-3) for 16 h, then nanoblades were added and DNA extraction was performed 24 h post-transduction. **(C)** CD34^+^ cells transduced with MLV or HIV nanoblades co-pseudotyped with BaEV- and VSVG-gps (B + V). Electrophoresis gel of the PCR product using WASFw/WASRv primers with the % gene-editing indicated for each lane (left) and summarizing graph showing percentage of gene editing (right) (MLV B + V *n* = 7, HIV B + V *n* = 6; student *t*-test, n.s., not significant). **(D)** Survival of CD34^+^ cells 72 h post-incubation with nanoblades as determined by propidium iodide staining and flow cytometry analysis (left graph) and CFC frequencies of CD34^+^ cells incubated for 16 h with or without MLV or HIV nanoblades and upon differentiation for 14 days into myeloid lineages (means ± SD MLV B + V *n* = 3, HIV B + V *n* = 3; n.s., not significant). **(E)** Surface staining of CD34^+^ cells for CD38 and CD90 at day 1 or 3 post MLV-nanoblade incubation (+NB) compared to controls (–NB). These data are representative of *n* = 2. **(F)** Sequence decomposition of INDEL events. PCR indicated DSBs at gRNA 301 or 305 target loci simultaneously by exon 1 drop-out (647 bp band; Figure 2B). Sanger sequencing of these gRNA 301 or 305 loci in the residual 811 bp PCR was subjected to ICE analysis which revealed on-target INDEL frequencies at gRNA 301 or 305 target loci individually.

In contrast to RNP electroporation, nanoblades transfer Cas9/gRNA complexes by a mild intervention, equivalent to enveloped pseudotyped retroviral vectors and might therefore be less toxic. Firstly, we transduced the CD34^+^ K562 cell line with equivalent amounts of MLV and HIV nanoblades. For K562 cells we obtained up to 65% deletion of the WAS gene using MLV nanoblades and up to 50% with HIV nanoblades (Figure 5A). In parallel, the CD34^+^ cells were pre-stimulated with a cytokine cocktail for 16 h and then incubated with the same doses of nanoblades as used for K562 cells; 24 h later we checked the efficiency of gene editing by PCR using the WASFw and WASRv primer pair (Figures 2B, 5B). For primary human CD34^+^ cells, we observed on average 35% deletion with both nanoblade systems upon a short incubation time (Figure 5C). Gene editing efficiency in CD34^+^ cells is thus only two-fold lower than that obtained in the K562 cell line (Figure 5B). Importantly, viability of human CD34^+^ cells was not at all affected by incubation with nanoblades (Figure 5D). Additionally, when the CD34^+^ cells treated with nanoblades or not were differentiated into myeloid lineages, no differences in CFC frequencies were detected (Figure 5D). To verify if the editing by nanoblades had an impact on CD34+ cell composition we performed a surface staining for CD34, CD38, and CD90. No differences in the percentage of the most primitive HSCs (CD34^+^CD38^−^CD90^+^) between untreated and nanoblade-treated CD34+ cells were detected. Thus, no skewing of the CD34^+^ subpopulation was induced (Figure 5E). As already mentioned, PCR analysis on genomic DNA only revealed the gene editing when both guide RNAs introduced DSBs simultaneously. However, each gRNA by itself might have induced additional DSBs at their respective target site. These events might also result in frameshift and thus knock-out of the WAS reading frame. Therefore, we separated the 811 bp PCR band from the 647 bp band (cut by both gRNAs) (Figure 5C). The 811 bp PCR product allowed to us to identify the single DSB events that occurred in addition to double DSB at either target site (gRNA 301 and −305). Both gRNA target sequences were separately subjected to Sanger sequencing using adapted primers and the resulting chromatographs were analyzed by ICE and TIDE to estimate the INDEL frequency at each gRNA-target site.

ICE analysis showed INDEL frequencies of up to 27% at the gRNA-301 target site and interestingly the most prevalent deletion consisted of 1 bp insertion (16%) resulting in a frameshift (Figure 5F). No additional INDELs were found at the gRNA-305 target site (Figure 5F). The same analysis was performed for 293T cells incubated with nanoblades, resulting in 60% DSBs at both gRNA targets simultaneously (Figure 2C). ICE analysis of the additional single site INDELs in 293T cells at gRNA-301 target revealed 71% INDEL frequency while at gRNA-305 target INDEL frequencies were much lower reaching only 1% (Supplementary Figures 4A,B) in accordance with the differential editing efficiency obtained in CD34^+^ cells for both gRNA-target sites (Figure 5F).

Although cleavage occurs with highest efficiency at on-target sites which are complementary to the gRNA protospacer domain, DSB could occur at loci with one or more mismatched bp compared to the on-target sites (gRNA 301 and 305). These events are called off-target effects and should be reduced to the minimum in gene therapy applications of genome-editing tools. We identified these sites for both gRNAs 301 and 305 (Supplementary Tables 1, 2). Firstly, we PCR-amplified some of the off-target sequences and performed the Surveyor assay to reveal possible mismatches that are recognized and cut by the T7 endonuclease. No off-target gene editing was revealed at the analyzed off-target sequences (Supplementary Figure 5). Since the Surveyor assay will not detect off-target cleavage < 5% (Sentmanat et al., 2018), we subjected the PCR products of off-target −2, −10 and −12 to Sanger sequencing and ICE analysis no off-target cleavage (INDELs) was detected using ICE analysis. TIDE analysis for the same off-target sites reported 1–3% total editing efficiency, although none of these predicted editing values were significant (*p* > 0.001) (Supplementary Figure 6) confirming that no off-target editing occurred.

### “Nanoblades” Loaded With Cas9/sgRNA Ribonucleoproteins Combined With AAV6 Encoding for Donor DNA, Allowed Efficient Knock-in in Human CD34^+^ Cells

**Figure 6:**
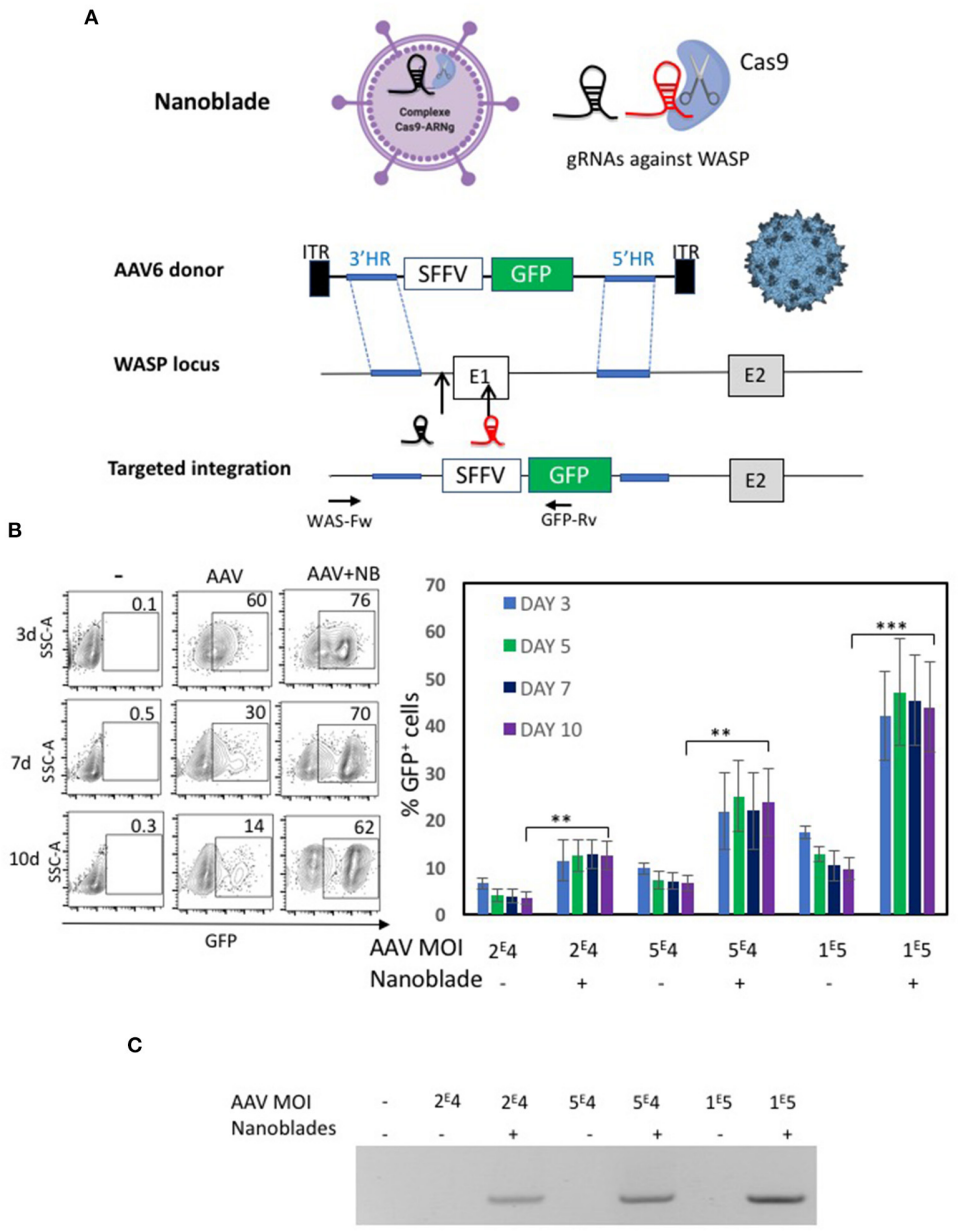
Evaluation of nanoblades combined with rAAV6 encoding donor DNA for WAS gene locus knock-in. **(A)** Schematic representation of the nanoblades loaded with Cas9 and two sgRNA directed to the WAS locus (indicated before and inside the first exon) and the rAAV6 genome carrying the donor DNA for homologous recombination (HR); 3' HR and 5'HR arms are indicated. An expression cassette with GFP under control of the SFFV promoter was inserted between the HR arms. Targeted integration is represented with primer positions indicated used to confirm specific integration (WAS-Fw and GFP-Rv**)**. **(B)** K562 cells were treated with rAAV6 vector alone or rAAV6 combined with MLV based nanoblades. A representative flow cytometry plot for GFP^+^ K562 cells is shown for day 3, 5, 7, and 10 post-treatment (left). A summary of the results is shown in the graph on the right (means ± SD; *n* = 4; student *t*-test, **p < 0.01, ***p < 0.001). MOIs for rAAV are indicated. **(C)** K562 genomic DNA at day 12 post-treatment was isolated and integration was determined by PCR using WAS-Fw and GFP-Rv indicated in **A**. The gel is representative of *n* = 4.

**Figure 7:**
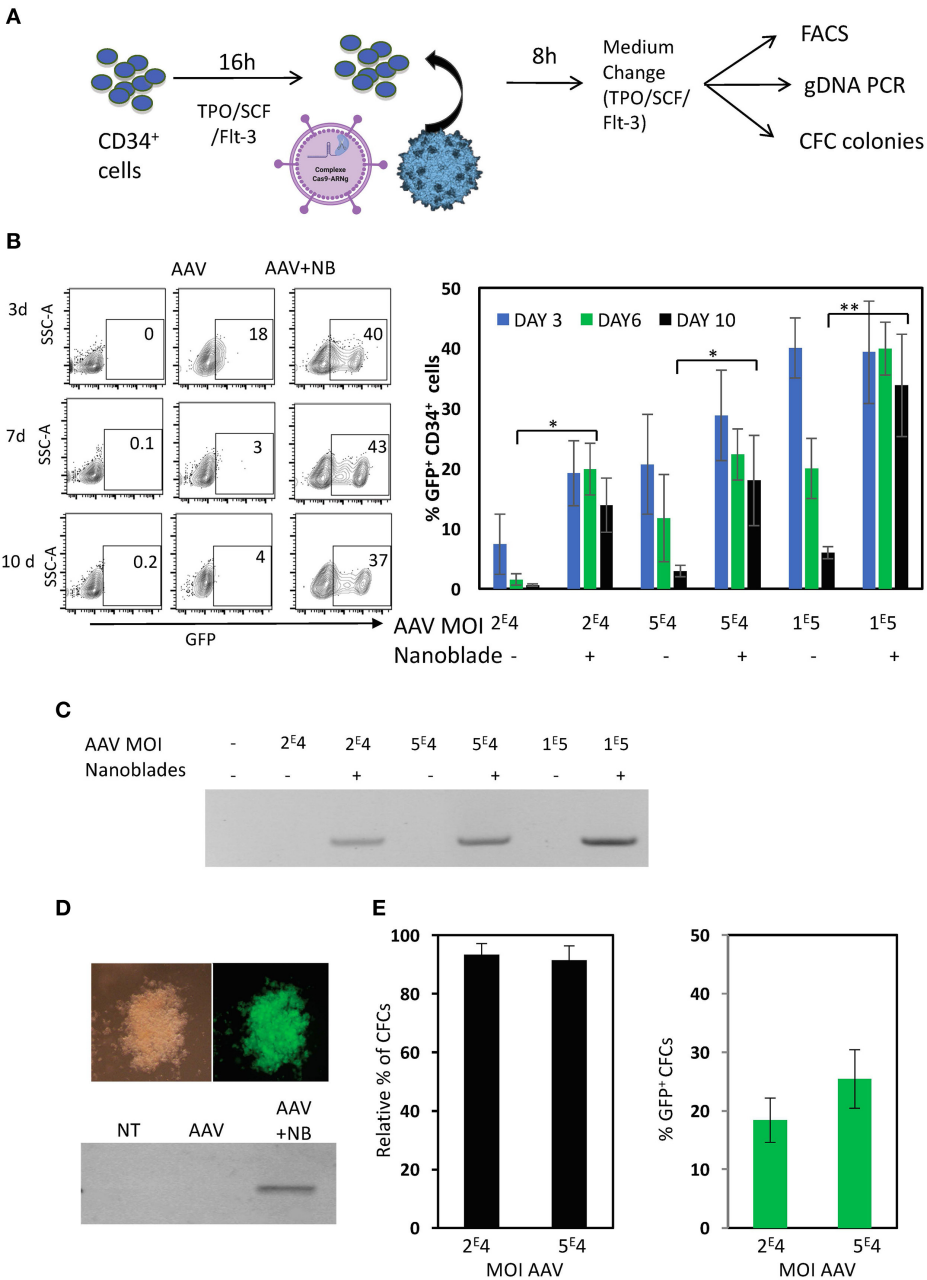
Nanoblades confer knock-in in human CD34^+^ cells when combined with rAAV6 coding for the DNA donor cassette. **(A)** Schematic presentation of protocol used for CD34^+^ cells. CD34^+^ cells were pre-activated for 72 h with cytokines (TPO/SCF/Flt-3), then rAAV6 vector carrying the donor alone or combined with nanoblades were added and DNA extraction was performed after 24 h incubation, while flow cytometry analysis was performed on day 3, 6, and 10. CFC colonies were seeded after 24 h incubation with rAAV6 or rAAV6 + nanoblades and CFC colonies were counted and screened at 14 days for GFP expressing by flow cytometry. **(B)** A representative flow cytometry plot for GFP^+^ CD34^+^ cell is shown for day 3, 6, and 10 post-treatment with rAAV (MOI = 1 × 10^5^) or rAAV+ nanoblades (left). A summary of the results is shown in the graph on the right (means ± SD; *n* = 4; four independent CD34^+^ cell donors; student *t*-test, *p < 0.05, **p < 0.01). MOIs for rAAV are indicated. **(C)** Genomic DNA at day 10 post-treatment was isolated and integration was determined by PCR using WAS-Fw and GFP-Rv in Figure 6A. Data are representative of *n* = 4. **(D)** At 24 h post-treatment CD34^+^ cells were differentiated into myeloid colonies. At day 14 of differentiation into CFC colonies, colonies were screened for GPF expression and genomic DNA was isolated and integration was determined by PCR using WAS-Fw and GFP-Rv. Data are representative of *n* = 3. **(E)** Relative % of number of colonies for nanoblades + rAAV6 treated CD34^+^ cells compared to untreated CD34^+^ cells and % of GFP^+^ at day 14 of differentiation. MOIs for rAAV6 are indicated.

MLV nanoblades performed slightly better than HIV nanoblades for gene editing in CD34^+^ cells (Figure 5C), therefore we focused for knock-in experiments on the former ones. For knock-in of an expression cassette into the first exon of the WAS locus, rAAV6 vectors are considered ideal candidates since they allow high-level transduction into CD34^+^ cells and have been used as tools for donor DNA transfer (Bak et al., 2018; Kuo et al., 2018). We generated an rAAV6 ssDNA vector genome encoding the template for homologous recombination including 3' HR and 5'HR arms of the WAS gene at each side of exon 1 (Figure 6A). An expression cassette with GFP under control of the SFFV promoter was inserted between the HR arms. The positions of the gRNAs, associating with Cas9 and loaded into the nanoblades are indicated. The resulting targeted integration cassette in WAS locus is represented and the positions of the primers, which allow to confirm specific on-target integration are indicated (Figure 6A). First, K562 cells were treated with rAAV6 donor vector alone or rAAV6 combined with MLV-based nanoblades. Insertion of the donor template in the WAS locus resulted in a significantly higher percentage of GFP^+^ K562 cells with high MFI on day 3, 5, 7, and 10 post-treatment as compared to rAAV6 treatment in absence of nanoblades (Figure 6B). rAAV6 incubation alone showed a lower percentage GFP^+^ K562 gradually decreasing over time in agreement with the fact that rAAV6 vectors are not integrative. This was true for rAAV6 used at different MOIs (2E4 to 1E5 vector genomes). K562 genomic DNA was isolated at day 12 post-treatment and integration was confirmed by PCR using the WAS-Fw and GFP-Rv primer pair (Figure 6A), confirming genomic integration by homologous recombination when nanoblades were combined with rAAV6 donor vector (Figure 6C). As expected, incubation with rAAV6 alones did not lead to detectable on-target integration. Since efficient knock-in was demonstrated into the WAS locus of the K562 cell line, we pre-activated CD34^+^ cells and treated them with the same combination of nanoblades and rAAV6 as indicated in Figure 7A. Addition of increasing amount of rAAV6 in combination with constant amounts of nanoblades resulted in 15% of knock-in for rAAV6 at MOI = 2E4, 20% for MOI = 5E4, and 35% for MOI = 1E5 (Figure 7B, day 10). In accordance with results obtained for K562 cells, insertion of the donor template in the WAS locus results in high levels of GFP^+^ CD34^+^cells with high MFI on day 3, 6, and 10 post-treatment (Figure 7B), while rAAV6 treatment in absence of nanoblades, resulted in decreasing percentages of GFP^+^ CD34^+^ cells over time (Figure 7C) in accordance with the fact that no integration was detected of the donor cassette at the on-target site (Figure 7D). Though not toxic for K562 cells, rAAV6 is known to be toxic at higher MOIs in CD34^+^ cells. Therefore, CD34^+^ cells treated with rAAV6 donor + nanoblades or rAAV6 alone were 24 h post-treatment allowed to differentiate into myeloid colony forming cells (CFCs). No significant decrease in number of colonies was detected compared to untransduced CD34^+^ cells for nanoblades + rAAV6 at MOI 2E4 or 5E4 (Figure 7E). In addition, the percentage of GFP^+^ colonies was equivalent with the initial percentage of GFP^+^ CD34^+^ cells from which these vectors were derived (Figures 7E vs. B).

## Discussion

Here we have demonstrated that nanoblades derived from HIV and MLV VLPs can deliver the Cas9-gRNA ribonucleoproteins into human T, B cells and HSPCs in a transient and rapid manner without need for strong activation of these gene therapy targets, conserving thereby their phenotypes. The high-level efficiency achieved in these gene therapy targets relied on combined pseudotyping of the nanoblades with VSV-G and BaEV envelope glycoproteins, assuring high-level incorporation of Cas9 endonuclease associated with gRNA into these VLP-like structures.

Most importantly, like a retroviral transduction of these target cells, no significant toxicity is induced by incubation with the Cas9/gRNA-loaded nanoblades. This is of utmost importance for gene editing of HSPCs, which are few in number and are not as readily available as T and B cells and easily lose their “stem cell” character by prolonged cytokine stimulation (Ahmed et al., 2004). We achieved gene editing of up to 40% of the cell revealed DSBs at two different gRNA target sites simultaneously in CD34^+^ cells upon a brief incubation without toxic effect. This underlines the advantage of the nanoblade technology. Moreover, we revealed that an additional 27% of the CD34^+^ cells harbored INDELs at a single gRNA target site. Further, HSPCs incubated simultaneously with nanoblades and rAAV6 vector coding for the donor DNA template resulted in up to 30–40 % of stable expression cassette knock-in into the WAS gene locus, levels relevant for clinical HSC-based gene therapy. Indeed, combining nanoblades with rAAV6 has allowed to achieve high level homologous recombination in HSPCs and was dependent on the doses of rAAV6 added. We cannot exclude here that the knock-in is driven mainly by gRNA301 since this guide was more efficient in inserting DSBs than the second gRNA305 in CD34+ cells and 293T cells. However, we cannot distinguish between knock-in upon DSB of gRNA301 or/and gRNA305 since the homologous arms remove both target sites after homologous recombination and insertion into WASP gene locus. Up to now most research groups used Cas9/gRNA RNPs and rAAV6-mediated donor template delivery targeting the CCR5 locus with an efficiency of 11% (Bak et al., 2017). Other authors targeting the IL-2RG locus to correct SCID-X1 achieved 20% HSPCs knock-in without strong toxicity (Pavel-Dinu et al., 2019). For other targeted genomic loci an efficiency of 20–30% was achieved for a monogenic knock-in (De Ravin et al., 2017; Charlesworth et al., 2018; Kuo et al., 2018; Gomez-Ospina et al., 2019; Wagenblast et al., 2019). It is estimated that a minimum gene correction/modification of 10–30% in CD34^+^ HSPCs is required to obtain a therapeutic benefit in autologous HSC transplantation (Morgan et al., 2017). The nanoblade-mediated gene knock-in in HSPCs achieved an efficiency that becomes clinically relevant opening avenues for multiple genetic diseases and beyond.

Also in human B cells gene editing was achieved using Cas9/gRNA RNPs with high efficiency (Johnson et al., 2018; Wu et al., 2018; Moffett et al., 2019). However, most of these studies though use co-culture of B cells with feeder cells or strong activation cocktails and detailed information on toxicity induced by the electroporation is not clearly evaluated. Moreover, one study reported that immunoglobulin heavy chain locus knock-in using CRISPR/Cas9 in human B cell is quite challenging (Hartweger et al., 2019). Nanoblades provide a tool for mild introduction of the Cas9/gRNA complexes into primary B cells upon brief prestimulation and with little to no effect on B cell survival.

Different platforms are being used to deliver the CRISPR/Cas9-gRNA platform. Lattanzi *et al*. (Lattanzi et al., 2019) made a side by side comparison between the delivery systems for this gene editing machinery in HSPCs. They concluded that plasmid electroporation, though highly efficient for edited deletions of large genome DNA sequences, was very toxic to the HSPCs revealed by strong reduction of clonogenic potential. RNA-mediated delivery was less efficient for gene editing but associated with high cell toxicity. Delivery *via* lentiviral transduction was less toxic but gene editing levels in HSPCs were poor. Of importance, lentiviral transduction to deliver Cas9/gRNA will result in stable persistent expression of these components into primary cells such as T cells and HSCs. This is an interesting approach to knock out genes of interest in primary cells, however, persistent Cas9 expression is not desirable in a therapeutic setting since this might incite an immune response against edited cells and increase off-target editing (Hendel et al., 2015b; Yu et al., 2016; Cameron et al., 2017). Additionally, continued Cas9 expression can lead to cell cycle arrest (Knopp et al., 2018). Finally, RNP-mediated delivery is the method of choice currently for primary cells because it results in high genome editing and much less cytotoxic effect as compared to previous methods above. Gundry et al. (2016) evaluated the impact of Cas9/gRNA RNP electroporation into CD34^+^ cells. Although this method is less harmful to those previously mentioned and high-level gene editing was detected at 48 h post electroporation, 50% of the cells did not survive. This toxicity level in CD34^+^ cells was consistent with other reports using the same approach (Genovese et al., 2014; De Ravin et al., 2017; Charlesworth et al., 2018), while higher viability was achieved after optimizing several parameters (Patsali et al., 2019).

Still the aim was to optimize gene editing with minimal cell toxicity. The nanoblades combine actually the low to undetected toxicity of retroviral delivery (VLP) and the transient expression of Cas9/gRNA RNP-mediated gene editing. Indeed, nanoblades confer efficient NHEJ-mediated gene editing in HSPCs but also in T cells but not at expense of significant induced toxicity. Indeed, we demonstrated that gene editing was stable long term in cell lines (293T and K652) treated with nanoblades and did not induce cell death nor effect on proliferation over non-treated cells. In accordance, we did not detect a significant effect on cell survival and proliferation in nanoblade treated vs. untreated primary T cells, nor in their phenotype and gene editing upon long-term culture. We demonstrated this additionally for HSPCs by the fact that they showed equivalent differentiation into myeloid lineages in absence or presence of nanoblades. To evaluate lymphoid differentiation we reconstituted NOD/SCID gammaC–/– (NSG) mice with nanoblades treated CD34^+^ cells, which were successful in terms of humanization. However, when we isolated CD34^+^ from the BM and T cells from the spleen of these mice we did not confirm the 30% gene editing that was detected in the initial CD34^+^ cell population (data not shown). This is not surprising since WASP knock-out CD34+ cells will not engraft in NSG mice if the KNOCK-out is not > 90% since they have a selective disadvantage compared to WASP-positive CD34^+^ cells (Mani et al., 2009). Moreover, from clinical trials it became clear that the WAS gene therapy conferred selective proliferative advantage for WASP expressing T and B cells and probably also HSCs (Ferrua et al., 2020, JACI).

Another important concern in use of gene editing approaches is the risk of off-target genome editing at loci that are similar in sequence of the on-target site. We have previously shown in the context of a well characterized gRNA targeting the EMX1 locus that plasmid introduction of the different CRISPR/Cas9 components resulted in 6% off-target, while the nanoblades did not reveal off-target genome editing despite of 75% on-target efficiency (Mangeot et al., 2019). In accordance, we confirmed that for two gRNA targeted to different WAS loci loaded into nanoblades no significant cutting could be detected at off-target sequences, although a more extensive off-target analysis is warranted especially when moving forward to the clinic. Equivalent to RNP transfection this is probably due to short and transient expression of the Cas9 complexed with the gRNAs.

Although corrections of β-hemaglobinopaties by gene-editing using HR were achieved in HSCs (Dever et al., 2016; Pavani et al., 2020), NHEJ might offer alternative correction strategies for gene therapy of β-thalassemia and Sickle cell disease. In their most common application, those NHEJ-mediated editing strategies efficiently disrupt disease modifiers or the β-globin locus (Cavazzana et al., 2017), resulting in therapeutic re-activation of fetal γ-globin genes, which is normally shut down in adults. Disruption of disease modifiers, such as of the γ-globin repressor BCL11A, can achieve high-level induction of γ-globin (Wu et al., 2019) in HSPCs by a single CRISPR/Cas9 RNP. Alternatively, disruption of the β-globin locus can be used to remove repressor elements of γ-globin, and in particular mimicking naturally occurring large deletions on the β-globin locus by a double-DSB strategy may achieve efficient γ-globin induction (Antoniani et al., 2018). Here we added to existing functional data for nanoblades by obtaining high-level genomic deletions in the WAS locus with nanoblades carrying two different gRNAs, which taken together validates nanoblades as efficient vectors for both the single- and double-DSB strategies outlined above.

Another monogenic disease that would benefit from nanoblade-mediated correction of HSPCs is the bone marrow failure, Fanconi Anemia (FA). HSPC-based gene therapy is very attractive treatment for FA because corrected stem cells have a selective advantage (Rio et al., 2017). This becomes clear by several different observations. Some FA patients acquired naturally correcting mutations in HSPCs, which led to expansion of these reverted clones and restoration of normal hematopoiesis (Gross et al., 2002; Mankad et al., 2006). Rio et al. achieved the same correction in FA HSPCs using an *ex vivo* lentiviral gene addition approach in a preclinical model recapitulating the expansion of reverted FA HSCs (Rio et al., 2017), which 2 years later was confirmed in the first HSC-LV-based trial for FA conducted without conditioning (Rio et al., 2019). However, since FA patients exhaust their HSCs and are easily induced into apopotosis by *ex vivo* culture and transduction procedures, HSC-based gene therapy is challenging (Verhoeyen et al., 2017). For this particular disease a corrective gene editing strategy would offer huge advantages since: (1) NHEJ repair upon gene-editing is increased in FA cells (Du et al., 2016); (2) NHEJ is the preferred mechanism of repair of DSBs in resting cells (Naka and Hirao, 2011) avoiding pre-stimulation of FA HSCs and avoiding cell death. Though NHEJ normally is used for producing knock-outs in cells by insertions or deletions, the same pathway can be utilized to create an INDEL next to an FA mutation leading to correction of FA phenotype. Roman-Rodriguez et al. (2019) therefore electroporated a pre-selected gRNA associated with Cas9 as RNPs into HSCs from FA patients and achieved correction of FA HSCs confirmed by their proliferative advantage. The introduction of the same Cas9/gRNA complex using nanoblades might allow to correct FA HSCs with even lower toxicity than RNP electroporation.

Three other groups have developed CRISPR/Cas9 vehicles that resemble the nanoblades described here. Knopp et al. (2018) replaced in an MLV retroviral particle, viral components with the MS2 phage packaging machinery to incorporate Cas9 mRNA and sgRNA into these VLPs, which were transiently delivered in multiple cell types. Gee et al. (2020) developed nanovesicles incorporating Cas9 protein and sgRNA, called NanoMedic. Using chemical induced dimerization Cas9 protein and the gRNAs encoding construct carrying a viral packaging signal both were incorporated into these nanovesicles. Indikova and Indik (2020) engineered lentivirus-based nanoparticles to co-package the U6-sgRNA template and the CRISPR/Cas9 fused with a virion-targeted protein Vpr (Vpr.Prot.Cas9), for simultaneous delivery to cells. The three systems were highly efficient for gene editing in cell lines and some in primary cells such as induced pluripotent stem cells. However, these transient CRISPR/Cas9 delivery systems were not evaluated for gene editing in human T, B and HSPCs.

The nanoblades represent a highly flexible platform for gene editing in primary hematopoietic cells and can be established easily in any laboratory. Firstly, only the plasmid coding for the gRNAs needs to be redesigned to target another genomic locus. Moreover, they can harbor two gRNAs as shown here but easily can incorporate multiple gRNAs to permit knock-out of multiple genes at once as we have demonstrated previously (Mangeot et al., 2019). Secondly, continuously, Cas9 proteins are improved to reduce off-target activity or increase efficiency and other targetable nucleases are identified e.g., Cpf1 nucleases, high fidelity Cas9, nickases, hyper-acurate Cas9 (Zetsche et al., 2015; Kleinstiver et al., 2016; Vakulskas et al., 2018). More recently, improved base editors (Webber et al., 2019) are becoming utilized for therapeutic applications (Osborn et al., 2020). All these new components for gene editing can readily be incorporated into nanoblades by fusing them to MLV or HIV gag proteins. Thirdly, since these nanoblades are derived from retroviral vectors, they can benefit from the same surface modifications, a process referred to as pseudotyping. We have previously shown that pseudotyping of HIV-derived vectors with heterologous envelopes such as baboon (BaEV) and measles virus (MV) gps, unlike the VSV-g envelope glycoprotein, allow more efficient fusion of viral membrane with the cell membrane. These BaEV and MV gp pseudotypes improved transduction of human T, B, NK cells, and HSCs (Girard-Gagnepain et al., 2014; Levy et al., 2016; Bernadin et al., 2019; Colamartino et al., 2019). We think that co-pseudotyping with VSV-G and BaEV gps on the nanoblades did not only allow more Cas9 incorporation as demonstrated but that the BaEV gp improved entry into these human blood cells as was true for their LV counterparts. VSV-G on the other hand will help the incorporation of heterologous proteins into the VLPs as it was shown that only VSV-G on its own formed “gesicules” and is able to embark high levels of protein as shown by us and our co-authors (Mangeot et al., 2011; Amirache et al., 2014). Therefore, VSV-G might help Cas9 incorporation into the nanoblades when co-expressed with BAEV gp. We also expect to have a higher incorporation of gRNA into the VSV-G+BAEV gp nanoblades since the incorporation of gRNA is Cas9 dependent as we have shown previously (Mangeot et al., 2019). In the case of MV gp pseudotyping this might even allow to improve nanoblade-mediated gene editing in the most primitive HSCs without stimulation since they allowed high level transduction when pseudotying LVs (Levy et al., 2017). More recently, LVs were engineered to specifically target hCD4^+^ or hCD8^+^ T cells through introduction of a scFv or a Designed Ankyrin repeat protein (DARPIN) directed against CD4 or CD8 epitopes into the measles virus glycoprotein H or the Nippa virus (NiV) glycoproteins G (Anliker et al., 2010; Bender et al., 2016). These CD4-MV and CD8-MV retargeted vectors showed, respectively, exclusive transduction into the CD4^+^ or CD8^+^ subset of hT cells *in vivo* in humanized NSG mice (Zhou et al., 2012, 2015). Additionally, transductional targeting of B cells and HSCs was achieved by direct inoculation of CD19-MV LVs or CD133-MV LVs, respectively into the bloodstream of humanized NSG mice (Kneissl et al., 2013; Brendel et al., 2015). Importantly, a single administration of CD8NiV-LVs encoding a CD19-CAR in the blood stream of human CD34+ humanized NSG mice generated CD19-CAR expressing CD8 T cells *in vivo*, which led to the depletion of the CD19^+^ B cells from all hematopoietic tissues (Pfeiffer et al., 2018). Thus, pseudotyping nanoblades with these retargeted envelopes gps might open the way to *in vivo* gene editing avoiding a costly *ex vivo* procedure. Finally, since the nanoblades are derived from retroviral vectors such as MLV or LV, scaling up of nanoblade production for clinical translation will be able to rely on some of the existing facilities and new production processes such as the CliniMACs Prodigy (Miltenyi Biotech, Germany) already available for MLV and LV vectors. Though adaptations will be needed this might speed up clinical translation of these new gene editing tools.

## Data Availability Statement

The raw data supporting the conclusions of this article will be made available by the authors, without undue reservation.

## Author Contributions

AG-G, MA, OB, FF, SP, GF, and AM-T designed, performed experiments, and analyzed results. FM and KB shared the WAS donor cassette and helped with design of the WAS gRNAs and off-target site identification. PM and CC cloned the HIV-derived nanoblade system. RG and SG performed the analysis for on-target and off-target sequences. EA cloned the donor and produced the rAAV6-donor vector batch. F-LC discussed results and commented the manuscript. ER and PM shared the MLV nanoblade system and co-designed experiments. EV supervised the work, designed, and performed experiments, discussed results and wrote the manuscript. AK made substantial contributions to the acquisition, analysis, or interpretation of data for the work during the revision process. All authors contributed to the article and approved the submitted version.

## Conflict of Interest

PM and ER are named as inventors on a patent relating to the Nanoblades technology [patent applicants: Institut National de la Sante et de la Recherche Medicale (INSERM), Centre National de la Recherche Scientifique (CNRS)], Ecole Normale Superieure de Lyon, Universite Claude Bernard Lyon 1, Villeurb-Anne Cedex; application number: WO 2017/068077 Al; patent status: published, 27th April 2017. The remaining authors declare that the research was conducted in the absence of any commercial or financial relationships that could be construed as a potential conflict of interest.
