# Supplementary material for: Baboon Envelope Pseudotyped “Nanoblades” Carrying Cas9/gRNA Complexes Allow Efficient Genome Editing in Human T, B, and CD34+ Cells and Knock-in of AAV6-Encoded Donor DNA in CD34+ Cells
Source: Front Genome Ed. 2021 Feb 9;3:604371. doi: 10.3389/fgeed.2021.604371 (PMC8525375; doi:10.3389/fgeed.2021.604371)
Supplement: Supplementary file 1 [file Presentation_1.PPTX]

## Slide 1
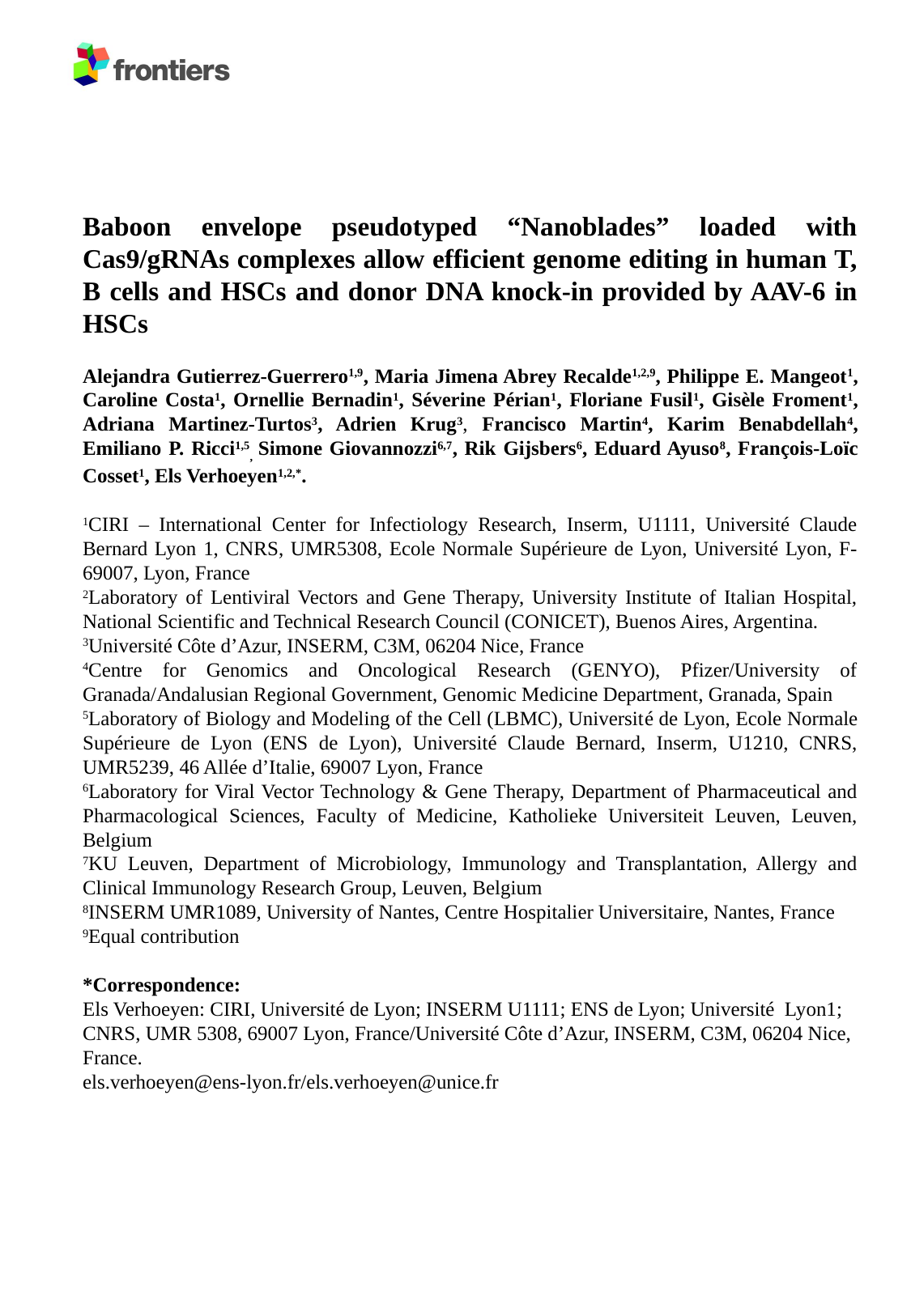

Baboon envelope pseudotyped “Nanoblades” loaded with Cas9/gRNAs complexes allow efficient genome editing in human T, B cells and HSCs and donor DNA knock-in provided by AAV-6 in HSCs
Alejandra Gutierrez-Guerrero1,9, Maria Jimena Abrey Recalde1,2,9, Philippe E. Mangeot1, Caroline Costa1, Ornellie Bernadin1, Séverine Périan1, Floriane Fusil1, Gisèle Froment1, Adriana Martinez-Turtos3, Adrien Krug3, Francisco Martin4, Karim Benabdellah4, Emiliano P. Ricci1,5, Simone Giovannozzi6,7, Rik Gijsbers6, Eduard Ayuso8, François-Loïc Cosset1, Els Verhoeyen1,2,*.
1CIRI – International Center for Infectiology Research, Inserm, U1111, Université Claude Bernard Lyon 1, CNRS, UMR5308, Ecole Normale Supérieure de Lyon, Université Lyon, F-69007, Lyon, France
2Laboratory of Lentiviral Vectors and Gene Therapy, University Institute of Italian Hospital, National Scientific and Technical Research Council (CONICET), Buenos Aires, Argentina.
3Université Côte d’Azur, INSERM, C3M, 06204 Nice, France
4Centre for Genomics and Oncological Research (GENYO), Pfizer/University of Granada/Andalusian Regional Government, Genomic Medicine Department, Granada, Spain
5Laboratory of Biology and Modeling of the Cell (LBMC), Université de Lyon, Ecole Normale Supérieure de Lyon (ENS de Lyon), Université Claude Bernard, Inserm, U1210, CNRS, UMR5239, 46 Allée d’Italie, 69007 Lyon, France
6Laboratory for Viral Vector Technology & Gene Therapy, Department of Pharmaceutical and Pharmacological Sciences, Faculty of Medicine, Katholieke Universiteit Leuven, Leuven, Belgium
7KU Leuven, Department of Microbiology, Immunology and Transplantation, Allergy and Clinical Immunology Research Group, Leuven, Belgium
8INSERM UMR1089, University of Nantes, Centre Hospitalier Universitaire, Nantes, France
9Equal contribution
*Correspondence:
Els Verhoeyen: CIRI, Université de Lyon; INSERM U1111; ENS de Lyon; Université Lyon1; CNRS, UMR 5308, 69007 Lyon, France/Université Côte d’Azur, INSERM, C3M, 06204 Nice, France.
els.verhoeyen@ens-lyon.fr/els.verhoeyen@unice.fr

## Slide 2
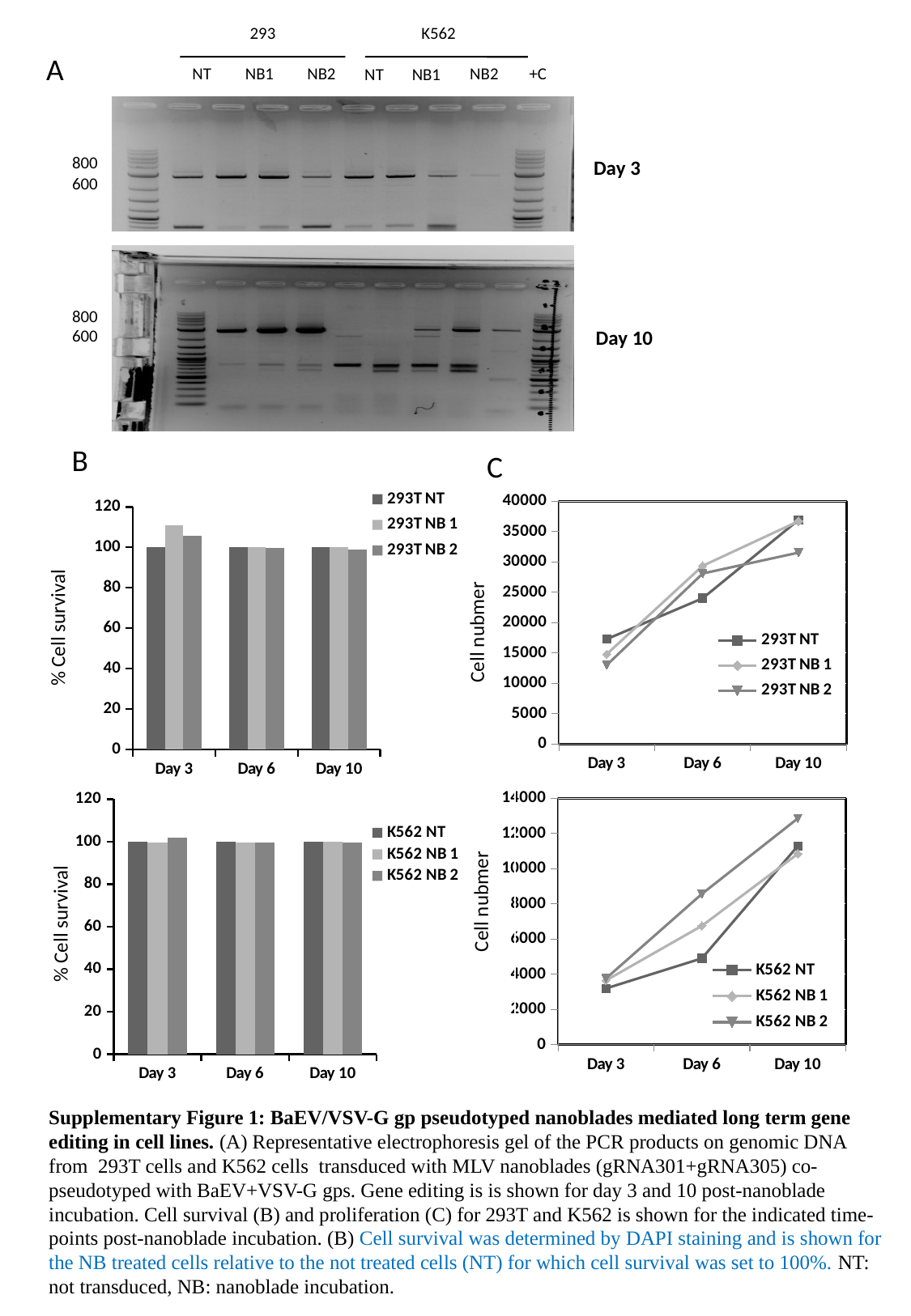

293
K562
NB2
NT
NB1
NB2
+C
NT
NB1
800
600
A
Day 3
800
600
Day 10
B
C
### Chart
| Category | 293T NT | 293T NB 1 | 293T NB 2 |
|---|---|---|---|
| Day 3 | 100.0 | 111.0584518167456 | 105.8451816745656 |
| Day 6 | 100.0 | 100.0 | 99.77553310886627 |
| Day 10 | 100.0 | 100.0 | 98.71977240398276 |
### Chart
| Category | 293T NT | 293T NB 1 | 293T NB 2 |
|---|---|---|---|
| Day 3 | 17275.0 | 14802.0 | 13008.0 |
| Day 6 | 24022.0 | 29369.0 | 28099.0 |
| Day 10 | 36927.0 | 36720.0 | 31542.0 |% Cell survival
Cell nubmer
### Chart
| Category | K562 NT | K562 NB 1 | K562 NB 2 |
|---|---|---|---|
| Day 3 | 3186.0 | 3640.0 | 3754.0 |
| Day 6 | 4896.0 | 6749.0 | 8565.0 |
| Day 10 | 11292.0 | 10842.0 | 12856.0 |
### Chart
| Category | K562 NT | K562 NB 1 | K562 NB 2 |
|---|---|---|---|
| Day 3 | 100.0 | 99.68976215098208 | 101.7580144777663 |
| Day 6 | 100.0 | 99.4913530010173 | 99.3896236012208 |
| Day 10 | 100.0 | 100.0 | 99.47643979057591 |Cell nubmer
% Cell survival
Supplementary Figure 1: BaEV/VSV-G gp pseudotyped nanoblades mediated long term gene editing in cell lines. (A) Representative electrophoresis gel of the PCR products on genomic DNA from 293T cells and K562 cells transduced with MLV nanoblades (gRNA301+gRNA305) co-pseudotyped with BaEV+VSV-G gps. Gene editing is is shown for day 3 and 10 post-nanoblade incubation. Cell survival (B) and proliferation (C) for 293T and K562 is shown for the indicated time-points post-nanoblade incubation. (B) Cell survival was determined by DAPI staining and is shown for the NB treated cells relative to the not treated cells (NT) for which cell survival was set to 100%. NT: not transduced, NB: nanoblade incubation.

## Slide 3
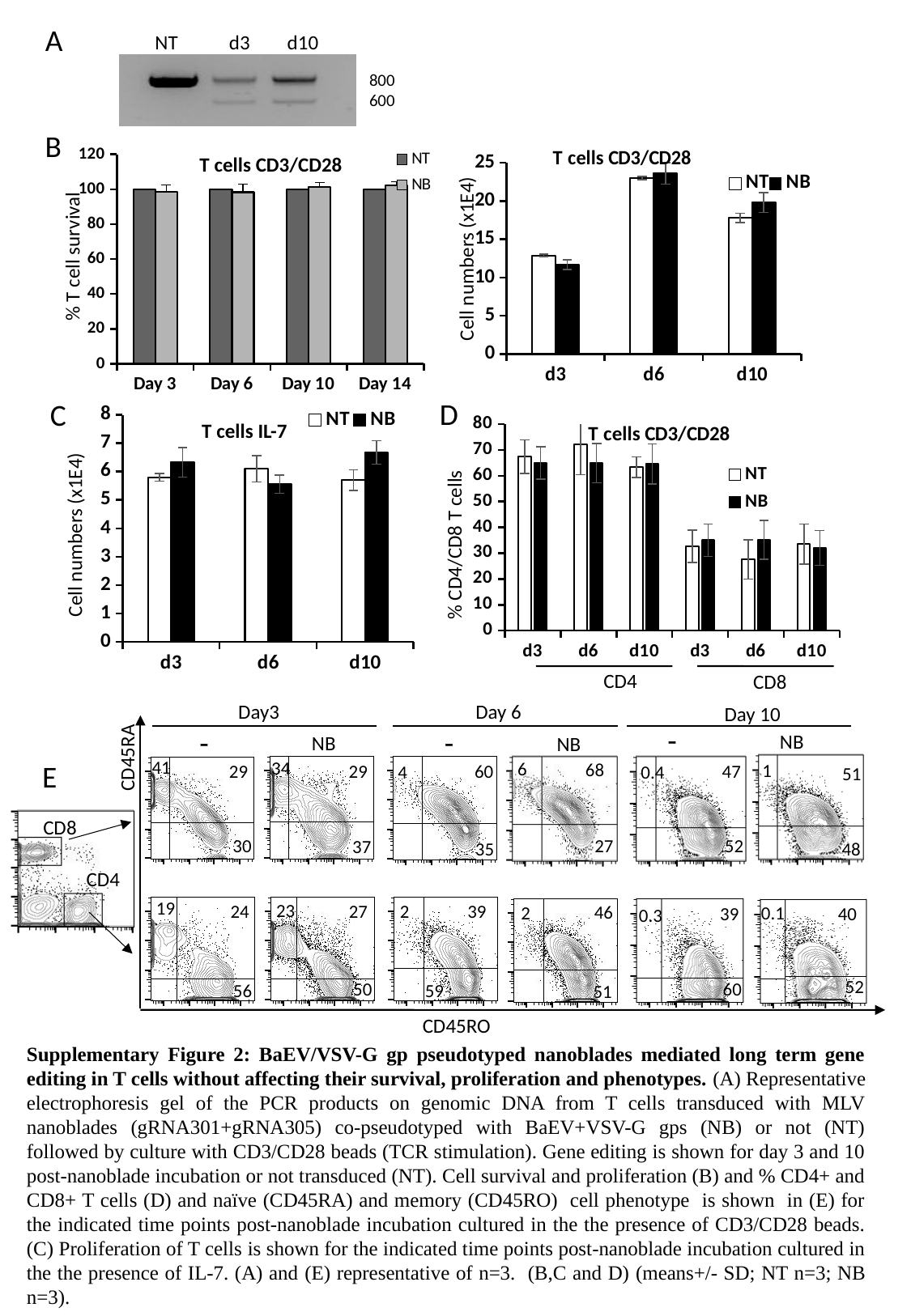

A
NT d3 d10
800
600
B
### Chart
| Category | NT | NB |
|---|---|---|
| d3 | 12.9 | 11.7 |
| d6 | 23.0 | 23.6 |
| d10 | 17.8 | 19.8 |
### Chart
| Category | NT | NB |
|---|---|---|
| Day 3 | 100.0 | 98.48374382217473 |
| Day 6 | 100.0 | 98.29214982813257 |
| Day 10 | 100.0 | 101.4580950107476 |
| Day 14 | 100.0 | 102.0375858676804 | T cells CD3/CD28
% T cell survival
Cell numbers (x1E4)
D
C
### Chart: T cells CD3/CD28
| Category | NT | NB |
|---|---|---|
| d3 | 67.36666666666665 | 65.0 |
| d6 | 72.16666666666667 | 64.86666666666665 |
| d10 | 63.35 | 64.60000000000001 |
| d3 | 32.63333333333333 | 35.0 |
| d6 | 27.56666666666667 | 35.13333333333333 |
| d10 | 33.5 | 32.03333333333333 |
### Chart
| Category | NT | NB |
|---|---|---|
| d3 | 5.8 | 6.324999999999989 |
| d6 | 6.1 | 5.558 |
| d10 | 5.7 | 6.673999999999999 |T cells IL-7
Cell numbers (x1E4)
% CD4/CD8 T cells
CD4
CD8
Day 6
Day3
Day 10
-
-
-
NB
NB
NB
CD45RA
41
34
6
E
68
1
47
29
60
29
4
0.4
51
CD8
30
27
52
37
48
35
CD4
19
23
24
2
27
39
46
2
0.1
40
39
0.3
52
50
60
56
59
51
CD45RO
Supplementary Figure 2: BaEV/VSV-G gp pseudotyped nanoblades mediated long term gene editing in T cells without affecting their survival, proliferation and phenotypes. (A) Representative electrophoresis gel of the PCR products on genomic DNA from T cells transduced with MLV nanoblades (gRNA301+gRNA305) co-pseudotyped with BaEV+VSV-G gps (NB) or not (NT) followed by culture with CD3/CD28 beads (TCR stimulation). Gene editing is shown for day 3 and 10 post-nanoblade incubation or not transduced (NT). Cell survival and proliferation (B) and % CD4+ and CD8+ T cells (D) and naïve (CD45RA) and memory (CD45RO) cell phenotype is shown in (E) for the indicated time points post-nanoblade incubation cultured in the the presence of CD3/CD28 beads. (C) Proliferation of T cells is shown for the indicated time points post-nanoblade incubation cultured in the the presence of IL-7. (A) and (E) representative of n=3. (B,C and D) (means+/- SD; NT n=3; NB n=3).

## Slide 4
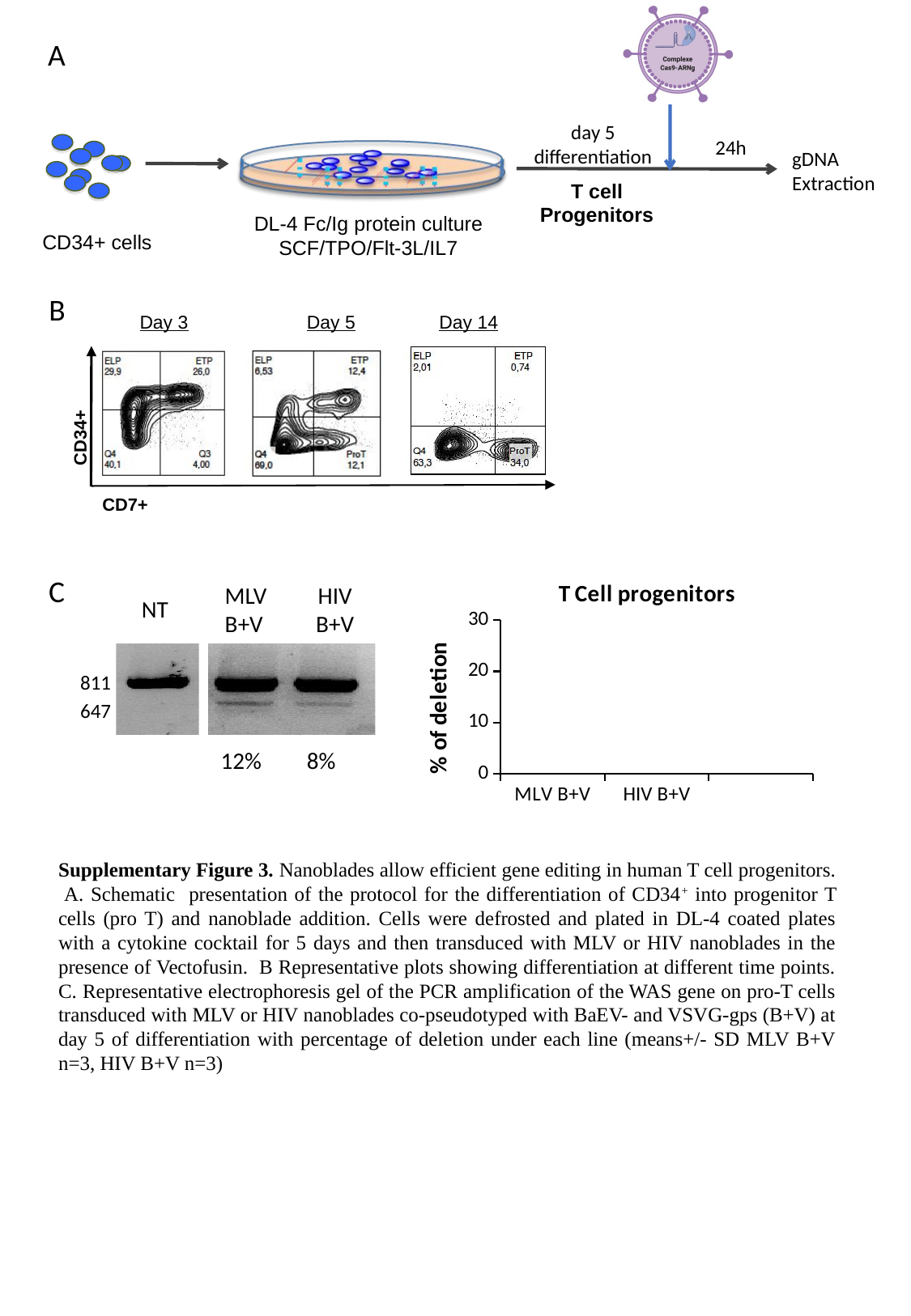

A
day 5
differentiation
24h
gDNA
Extraction
T cell
Progenitors
DL-4 Fc/Ig protein culture
SCF/TPO/Flt-3L/IL7
CD34+ cells
B
Day 3
Day 5
Day 14
CD34+
CD7+
### Chart: T Cell progenitors
| Category | |
|---|---|
| MLV B+V | 13.2 |
| HIV B+V | 9.5 |C
HIVB+V
MLV
B+V
NT
8%
811
647
12%
Supplementary Figure 3. Nanoblades allow efficient gene editing in human T cell progenitors. A. Schematic presentation of the protocol for the differentiation of CD34+ into progenitor T cells (pro T) and nanoblade addition. Cells were defrosted and plated in DL-4 coated plates with a cytokine cocktail for 5 days and then transduced with MLV or HIV nanoblades in the presence of Vectofusin. B Representative plots showing differentiation at different time points. C. Representative electrophoresis gel of the PCR amplification of the WAS gene on pro-T cells transduced with MLV or HIV nanoblades co-pseudotyped with BaEV- and VSVG-gps (B+V) at day 5 of differentiation with percentage of deletion under each line (means+/- SD MLV B+V n=3, HIV B+V n=3)

## Slide 5
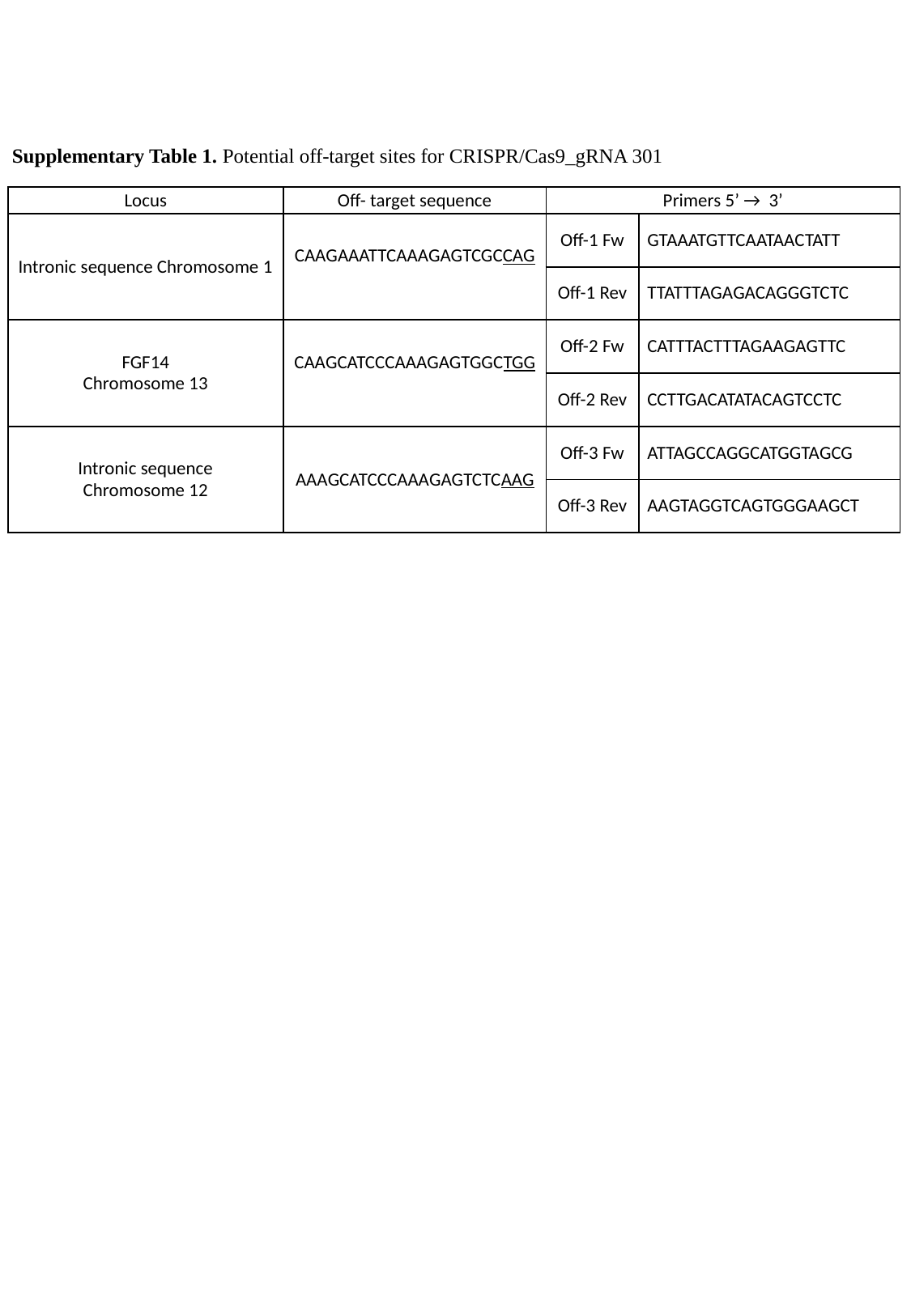

Supplementary Table 1. Potential off-target sites for CRISPR/Cas9_gRNA 301
| Locus | Off- target sequence | Primers 5’ → 3’ | |
| --- | --- | --- | --- |
| Intronic sequence Chromosome 1 | CAAGAAATTCAAAGAGTCGCCAG | Off-1 Fw | GTAAATGTTCAATAACTATT |
| | | Off-1 Rev | TTATTTAGAGACAGGGTCTC |
| FGF14 Chromosome 13 | CAAGCATCCCAAAGAGTGGCTGG | Off-2 Fw | CATTTACTTTAGAAGAGTTC |
| | | Off-2 Rev | CCTTGACATATACAGTCCTC |
| Intronic sequence Chromosome 12 | AAAGCATCCCAAAGAGTCTCAAG | Off-3 Fw | ATTAGCCAGGCATGGTAGCG |
| | | Off-3 Rev | AAGTAGGTCAGTGGGAAGCT |

## Slide 6
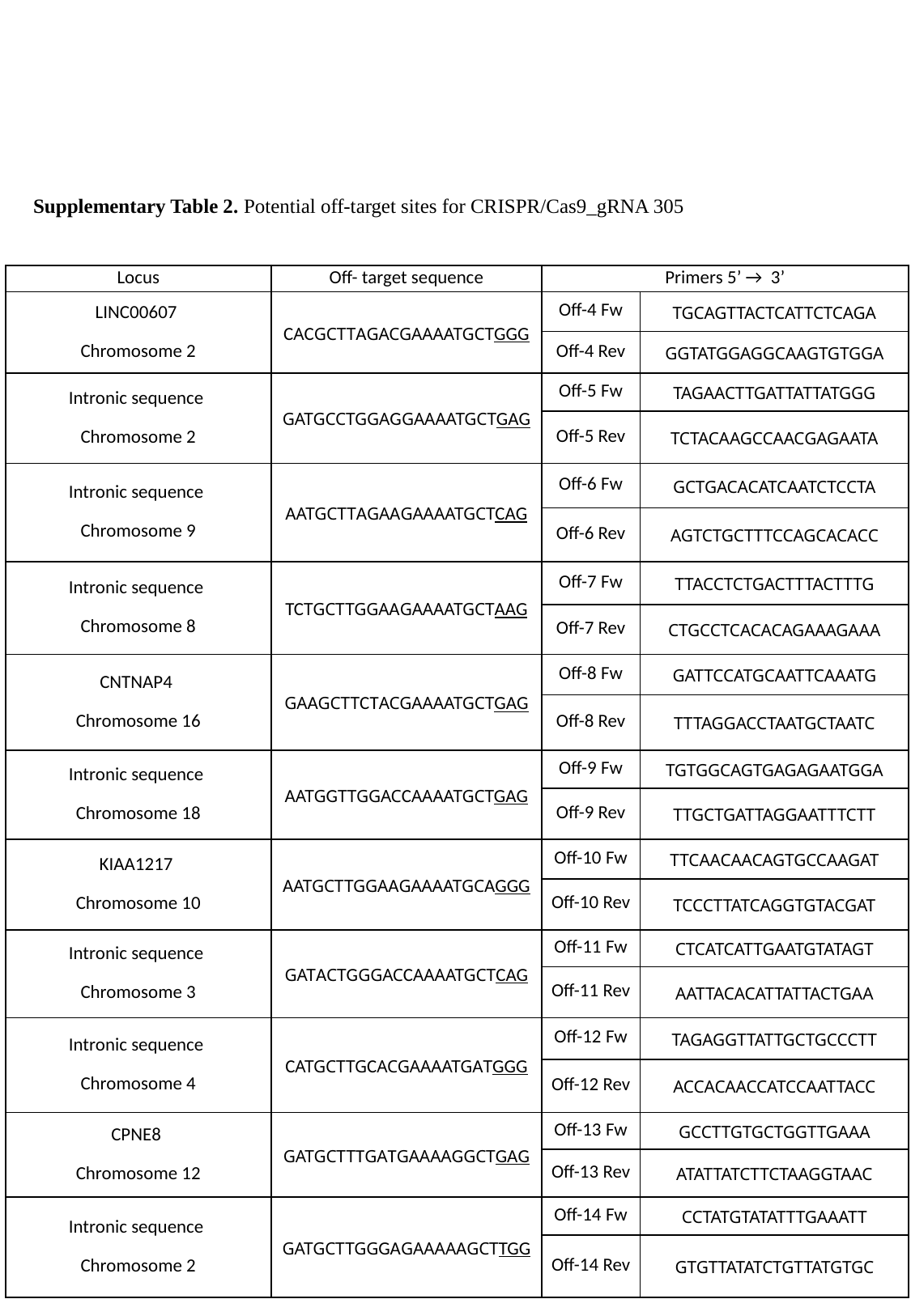

Supplementary Table 2. Potential off-target sites for CRISPR/Cas9_gRNA 305
| Locus | Off- target sequence | Primers 5’ → 3’ | |
| --- | --- | --- | --- |
| LINC00607 Chromosome 2 | CACGCTTAGACGAAAATGCTGGG | Off-4 Fw | TGCAGTTACTCATTCTCAGA |
| | | Off-4 Rev | GGTATGGAGGCAAGTGTGGA |
| Intronic sequence Chromosome 2 | GATGCCTGGAGGAAAATGCTGAG | Off-5 Fw | TAGAACTTGATTATTATGGG |
| | | Off-5 Rev | TCTACAAGCCAACGAGAATA |
| Intronic sequence Chromosome 9 | AATGCTTAGAAGAAAATGCTCAG | Off-6 Fw | GCTGACACATCAATCTCCTA |
| | | Off-6 Rev | AGTCTGCTTTCCAGCACACC |
| Intronic sequence Chromosome 8 | TCTGCTTGGAAGAAAATGCTAAG | Off-7 Fw | TTACCTCTGACTTTACTTTG |
| | | Off-7 Rev | CTGCCTCACACAGAAAGAAA |
| CNTNAP4 Chromosome 16 | GAAGCTTCTACGAAAATGCTGAG | Off-8 Fw | GATTCCATGCAATTCAAATG |
| | | Off-8 Rev | TTTAGGACCTAATGCTAATC |
| Intronic sequence Chromosome 18 | AATGGTTGGACCAAAATGCTGAG | Off-9 Fw | TGTGGCAGTGAGAGAATGGA |
| | | Off-9 Rev | TTGCTGATTAGGAATTTCTT |
| KIAA1217 Chromosome 10 | AATGCTTGGAAGAAAATGCAGGG | Off-10 Fw | TTCAACAACAGTGCCAAGAT |
| | | Off-10 Rev | TCCCTTATCAGGTGTACGAT |
| Intronic sequence Chromosome 3 | GATACTGGGACCAAAATGCTCAG | Off-11 Fw | CTCATCATTGAATGTATAGT |
| | | Off-11 Rev | AATTACACATTATTACTGAA |
| Intronic sequence Chromosome 4 | CATGCTTGCACGAAAATGATGGG | Off-12 Fw | TAGAGGTTATTGCTGCCCTT |
| | | Off-12 Rev | ACCACAACCATCCAATTACC |
| CPNE8 Chromosome 12 | GATGCTTTGATGAAAAGGCTGAG | Off-13 Fw | GCCTTGTGCTGGTTGAAA |
| | | Off-13 Rev | ATATTATCTTCTAAGGTAAC |
| Intronic sequence Chromosome 2 | GATGCTTGGGAGAAAAAGCTTGG | Off-14 Fw | CCTATGTATATTTGAAATT |
| | | Off-14 Rev | GTGTTATATCTGTTATGTGC |

## Slide 7
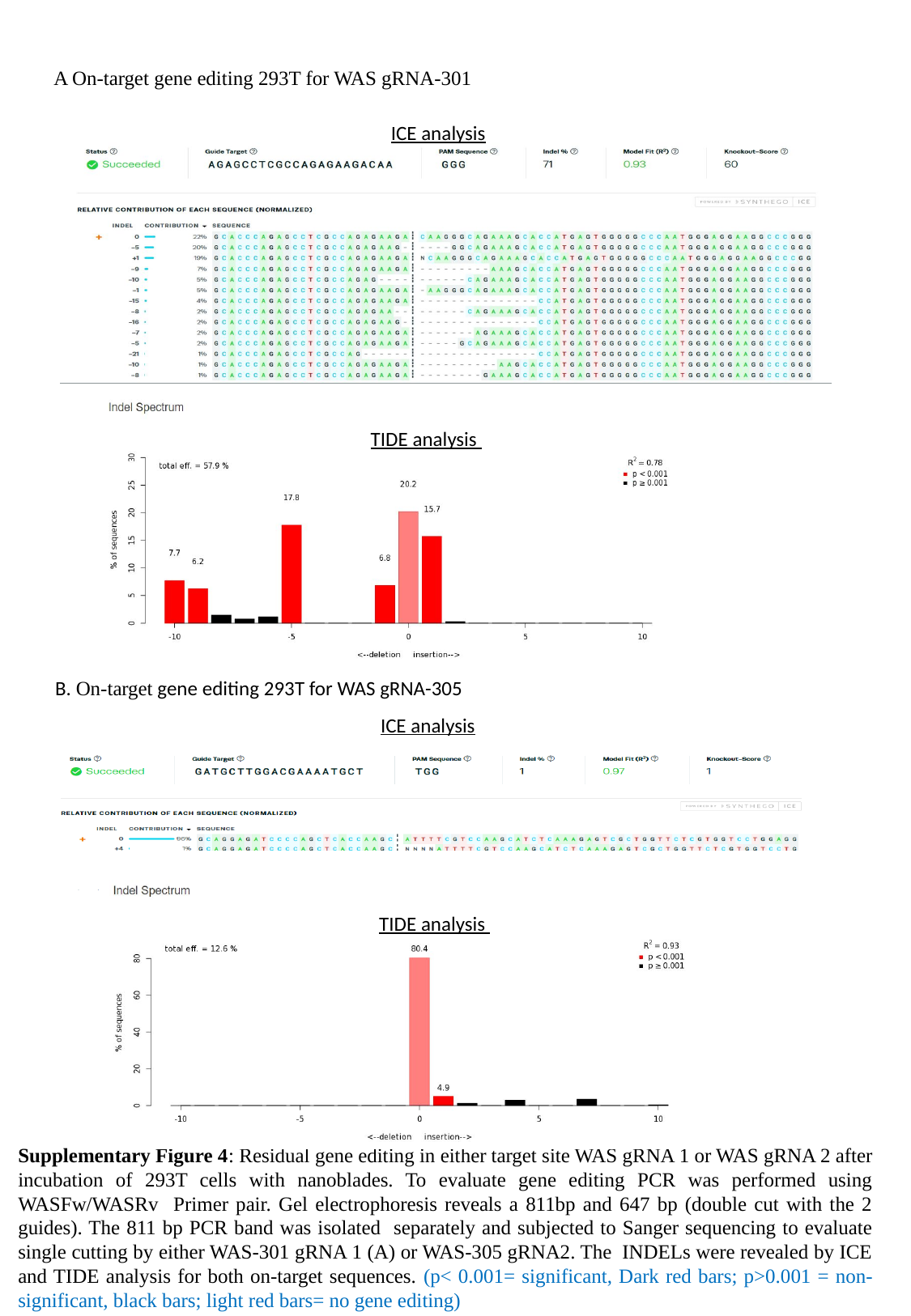

A On-target gene editing 293T for WAS gRNA-301
ICE analysis
TIDE analysis
B. On-target gene editing 293T for WAS gRNA-305
ICE analysis
TIDE analysis
Supplementary Figure 4: Residual gene editing in either target site WAS gRNA 1 or WAS gRNA 2 after incubation of 293T cells with nanoblades. To evaluate gene editing PCR was performed using WASFw/WASRv Primer pair. Gel electrophoresis reveals a 811bp and 647 bp (double cut with the 2 guides). The 811 bp PCR band was isolated separately and subjected to Sanger sequencing to evaluate single cutting by either WAS-301 gRNA 1 (A) or WAS-305 gRNA2. The INDELs were revealed by ICE and TIDE analysis for both on-target sequences. (p< 0.001= significant, Dark red bars; p>0.001 = non-significant, black bars; light red bars= no gene editing)

## Slide 8
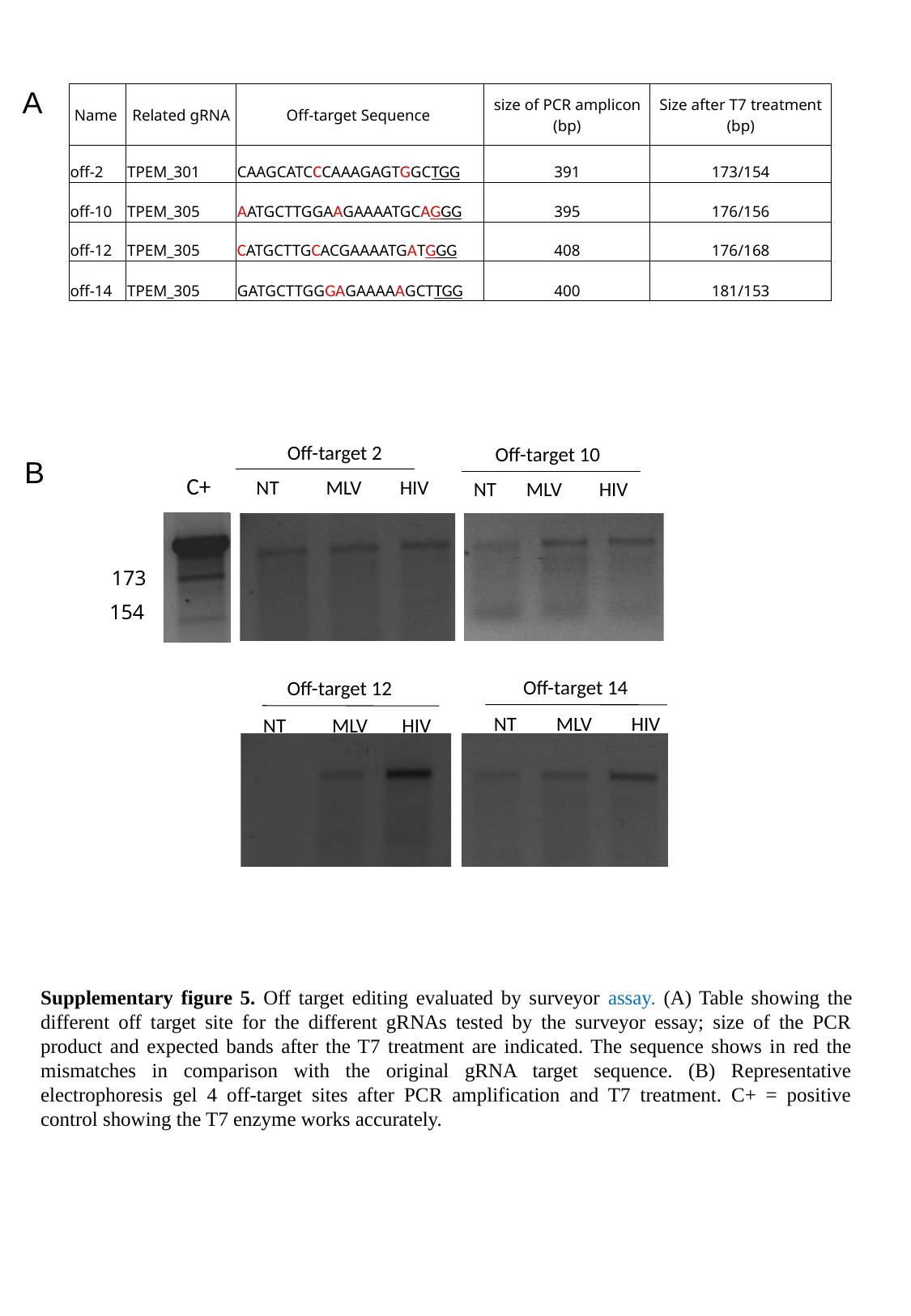

A
| Name | Related gRNA | Off-target Sequence | size of PCR amplicon (bp) | Size after T7 treatment (bp) |
| --- | --- | --- | --- | --- |
| off-2 | TPEM\_301 | CAAGCATCCCAAAGAGTGGCTGG | 391 | 173/154 |
| off-10 | TPEM\_305 | AATGCTTGGAAGAAAATGCAGGG | 395 | 176/156 |
| off-12 | TPEM\_305 | CATGCTTGCACGAAAATGATGGG | 408 | 176/168 |
| off-14 | TPEM\_305 | GATGCTTGGGAGAAAAAGCTTGG | 400 | 181/153 |
Off-target 2
Off-target 10
B
C+
NT
MLV
HIV
NT
MLV
HIV
173
154
Off-target 14
Off-target 12
NT
MLV
HIV
NT
MLV
HIV
Supplementary figure 5. Off target editing evaluated by surveyor assay. (A) Table showing the different off target site for the different gRNAs tested by the surveyor essay; size of the PCR product and expected bands after the T7 treatment are indicated. The sequence shows in red the mismatches in comparison with the original gRNA target sequence. (B) Representative electrophoresis gel 4 off-target sites after PCR amplification and T7 treatment. C+ = positive control showing the T7 enzyme works accurately.

## Slide 9
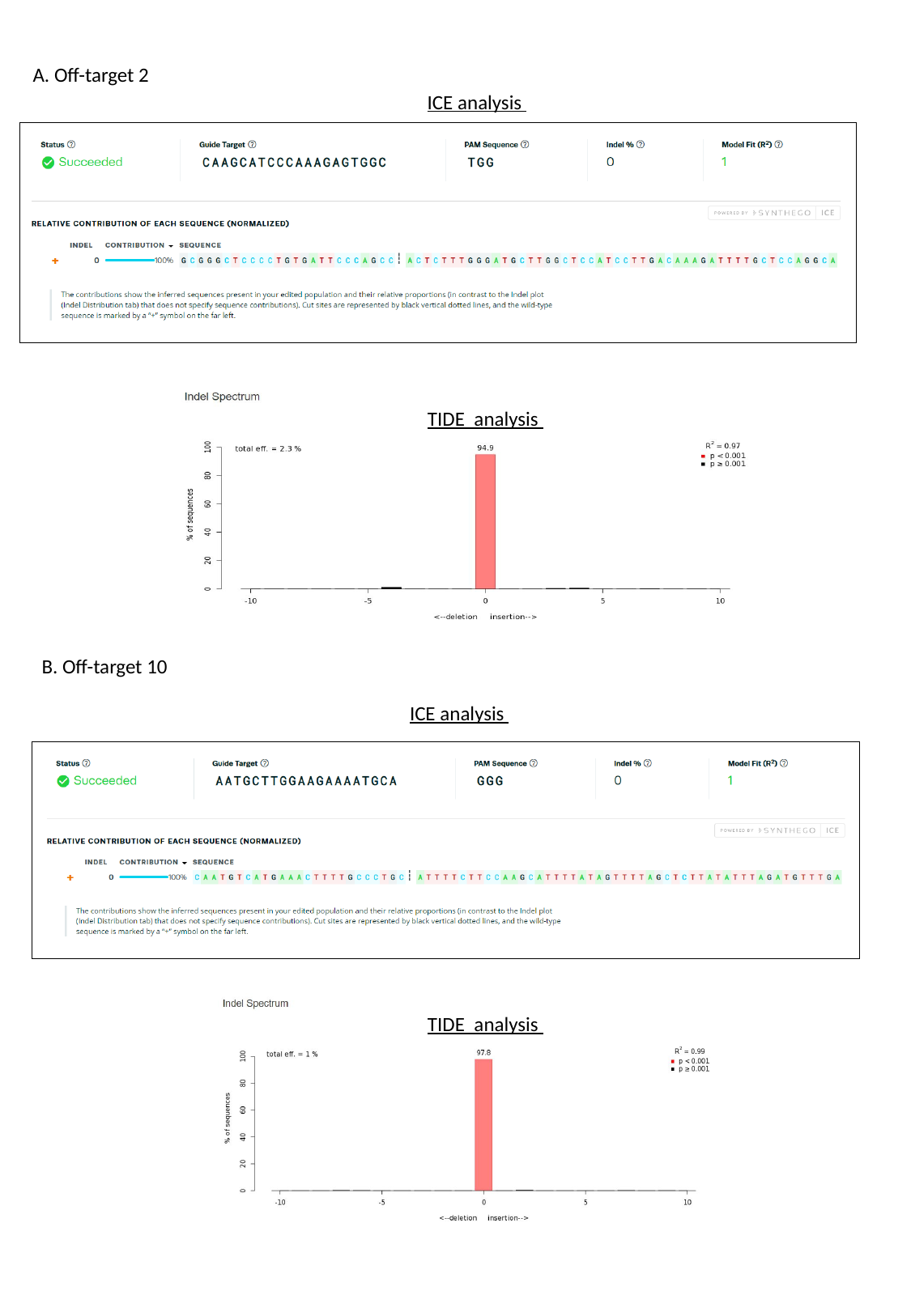

A. Off-target 2
ICE analysis
TIDE analysis
B. Off-target 10
ICE analysis
TIDE analysis

## Slide 10
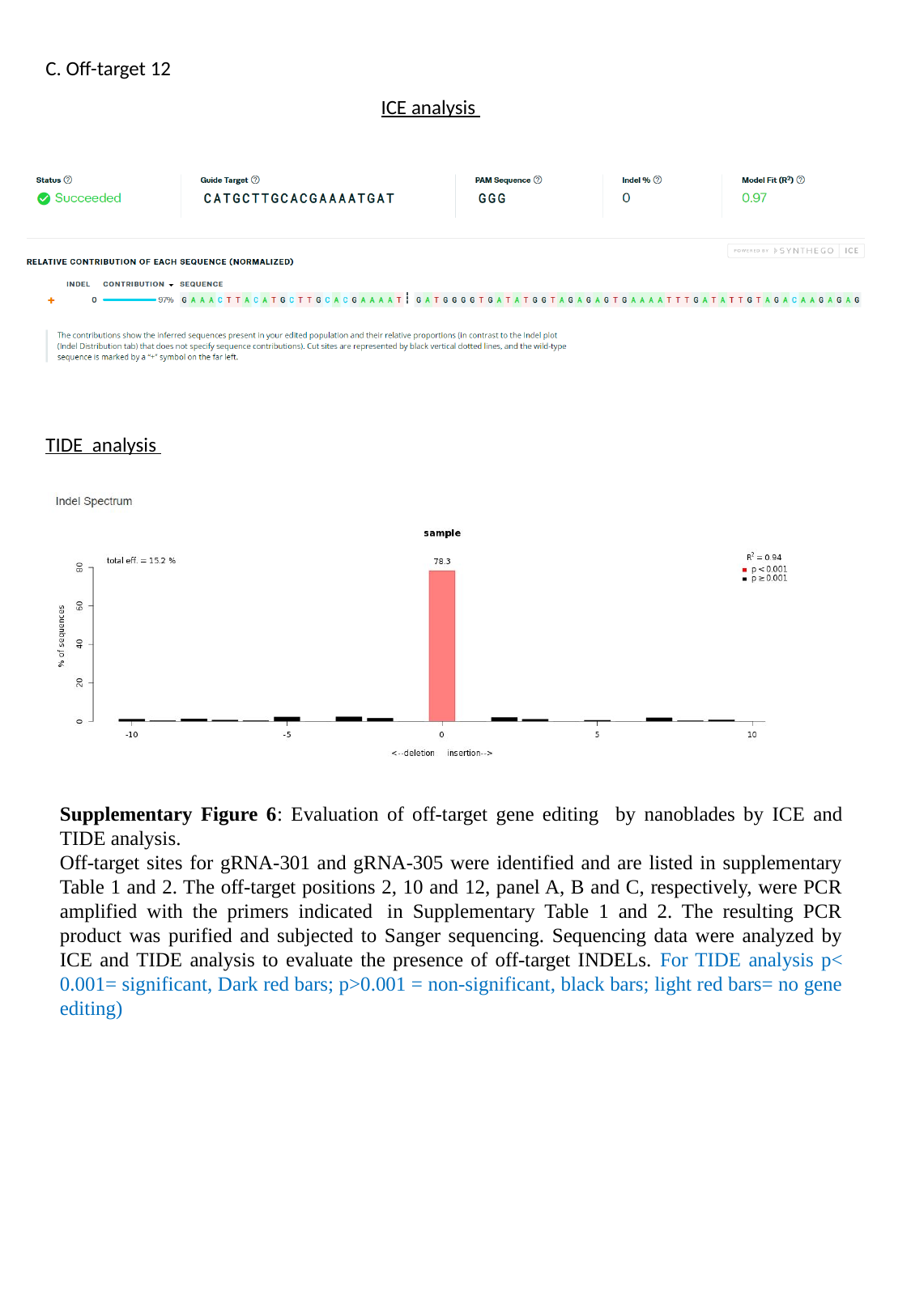

C. Off-target 12
ICE analysis
TIDE analysis
Supplementary Figure 6: Evaluation of off-target gene editing by nanoblades by ICE and TIDE analysis.
Off-target sites for gRNA-301 and gRNA-305 were identified and are listed in supplementary Table 1 and 2. The off-target positions 2, 10 and 12, panel A, B and C, respectively, were PCR amplified with the primers indicated  in Supplementary Table 1 and 2. The resulting PCR product was purified and subjected to Sanger sequencing. Sequencing data were analyzed by ICE and TIDE analysis to evaluate the presence of off-target INDELs. For TIDE analysis p< 0.001= significant, Dark red bars; p>0.001 = non-significant, black bars; light red bars= no gene editing)
