# Supplementary material for: Baboon Envelope Pseudotyped “Nanoblades” Carrying Cas9/gRNA Complexes Allow Efficient Genome Editing in Human T, B, and CD34+ Cells and Knock-in of AAV6-Encoded Donor DNA in CD34+ Cells
Source: Front Genome Ed. 2021 Feb 9;3:604371. doi: 10.3389/fgeed.2021.604371 (PMC8525375; doi:10.3389/fgeed.2021.604371)
Supplement: Supplementary file 2 [file Data_Sheet_1.PDF]

A. Humanisation NSG mice

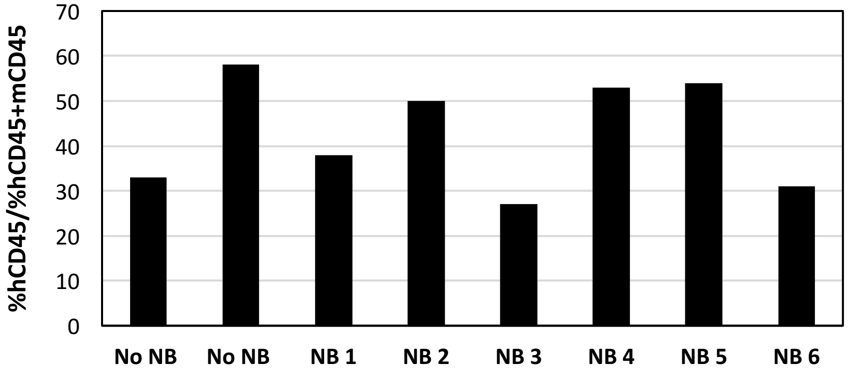

B. CD34+ bone marrow cells from humanized NSG

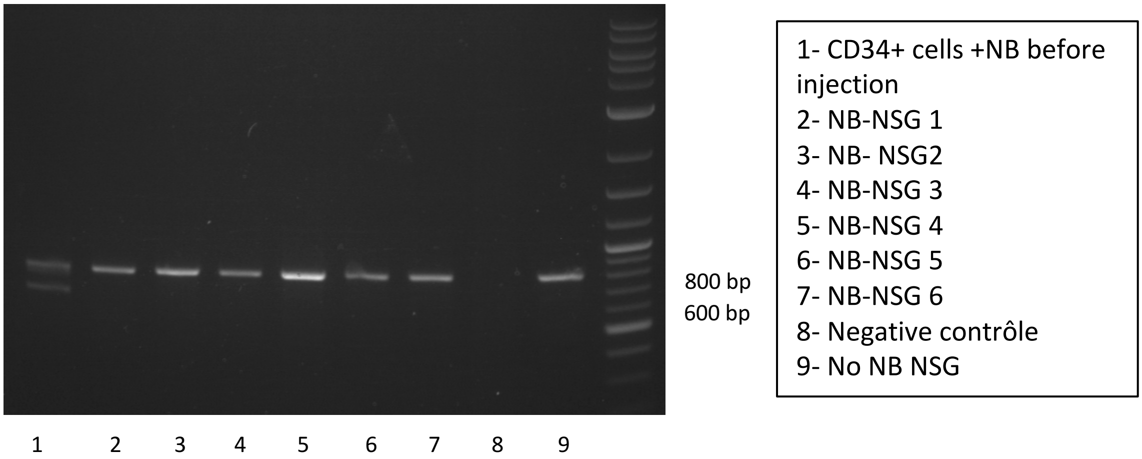

C. CD3+ spleen cells from humanized NSG mice

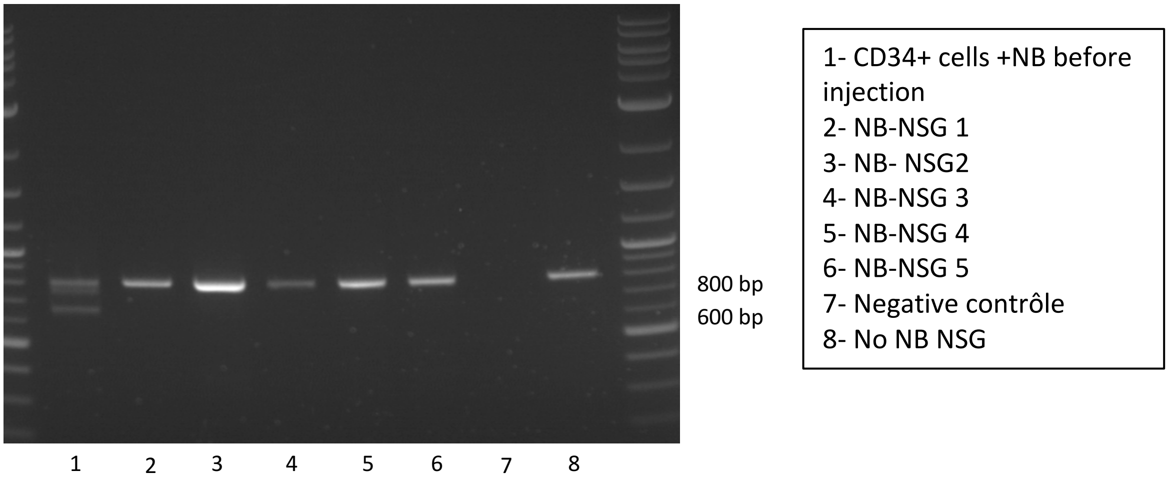

Figure A: **Gene edited WASP KO CD34+ cells have a selective disadvantage upon transplantation into NOD/SCIDgammaC-/- (NSG) mice**. NSG mice were injected with 2E5 hCD34+ cells treated with nanoblades g301+g305 (NB) or not of 8 hours. Upon 10 weeks of humanization (A), mice were sacrificed and hCD34+ cells were isolated from the BM and CD3+ T cells were isolated from the spleen. Verification of gene editing by PCR was performed for the hCD34+ cells (B) and hCD3+ cells. The input CD34+ cells treated with NBs were also verified for gene editing by PCR.
